# *Candida albicans FRE8* encodes a member of the NADPH oxidase family that produces a burst of ROS during fungal morphogenesis

**DOI:** 10.1371/journal.ppat.1006763

**Published:** 2017-12-01

**Authors:** Diego C. P. Rossi, Julie E. Gleason, Hiram Sanchez, Sabrina S. Schatzman, Edward M. Culbertson, Chad J. Johnson, Christopher A. McNees, Carolina Coelho, Jeniel E. Nett, David R. Andes, Brendan P. Cormack, Valeria C. Culotta

**Affiliations:** 1 Department of Biochemistry and Molecular Biology, Johns Hopkins University Bloomberg School of Public Health, Baltimore, Maryland, United States of America; 2 Departments of Medicine and of Medical Microbiology and Immunology, University of Wisconsin, Madison, Madison, Wisconsin, United States of America; 3 Department of Molecular Microbiology and Immunology, Johns Hopkins University Bloomberg School of Public Health, Baltimore, Maryland, United States of America; 4 Department of Molecular Biology and Genetics, Johns Hopkins University School of Medicine, Baltimore, Maryland, United States of America; University of Iowa, UNITED STATES

## Abstract

Until recently, NADPH oxidase (NOX) enzymes were thought to be a property of multicellularity, where the reactive oxygen species (ROS) produced by NOX acts in signaling processes or in attacking invading microbes through oxidative damage. We demonstrate here that the unicellular yeast and opportunistic fungal pathogen *Candida albicans* is capable of a ROS burst using a member of the NOX enzyme family, which we identify as Fre8. *C*. *albicans* can exist in either a unicellular yeast-like budding form or as filamentous multicellular hyphae or pseudohyphae, and the ROS burst of Fre8 begins as cells transition to the hyphal state. Fre8 is induced during hyphal morphogenesis and specifically produces ROS at the growing tip of the polarized cell. The superoxide dismutase Sod5 is co-induced with Fre8 and our findings are consistent with a model in which extracellular Sod5 acts as partner for Fre8, converting Fre8-derived superoxide to the diffusible H_2_O_2_ molecule. Mutants of *fre8*Δ/Δ exhibit a morphogenesis defect *in vitro* and are specifically impaired in development or maintenance of elongated hyphae, a defect that is rescued by exogenous sources of H_2_O_2_. A *fre8*Δ/Δ deficiency in hyphal development was similarly observed *in vivo* during *C*. *albicans* invasion of the kidney in a mouse model for disseminated candidiasis. Moreover *C*. *albicans fre8*Δ/Δ mutants showed defects in a rat catheter model for biofilms. Together these studies demonstrate that like multicellular organisms, *C*. *albicans* expresses NOX to produce ROS and this ROS helps drive fungal morphogenesis in the animal host.

## Author summary

We demonstrate here that the opportunistic human fungal pathogen *Candida albicans* uses a NADPH oxidase enzyme (NOX) and reactive oxygen species (ROS) to control morphogenesis in an animal host. *C*. *albicans* was not previously known to express NOX enzymes as these were thought to be a property of multicellular organisms, not unicellular yeasts. We describe here the identification of *C*. *albicans* Fre8 as the first NOX enzyme that can produce extracellular ROS in a unicellular yeast. *C*. *albicans* can exist as either a unicellular yeast or as multicellular elongated hyphae, and Fre8 is specially expressed during transition to the hyphal state where it works to produce ROS at the growing tip of the polarized cell. *C*. *albicans* cells lacking Fre8 exhibit a deficiency in elongated hyphae during fungal invasion of the kidney in a mouse model for systemic candidiasis. Moreover, Fre8 is required for fungal survival in a rodent model for catheter biofilms. These findings implicate a role for fungal derived ROS in controlling morphogenesis of this important fungal pathogen for public health.

## Introduction

Reactive oxygen species (ROS) including superoxide anion and hydrogen peroxide play diverse roles in biology. ROS can inflict severe oxidative damage to cellular components, but when carefully controlled, ROS can also be used to combat infection and act in cell signaling processes. A well-studied example of controlled ROS production involves NADPH oxidase (NOX) enzymes [1]. These heme and flavin containing enzymes use electrons from NADPH to reduce molecular oxygen to superoxide [1]. In macrophages and neutrophils, NOX enzymes generate bursts of superoxide in the extracellular milieu or phagolysosomal compartments to assault microbial pathogens. In non-immune cells, ROS from NOX enzymes are widely used in cell signaling pathways to promote growth, development and differentiation [1]. As membrane proteins, NOX enzymes can vectorially release superoxide inside the cell or extracellularly and in either case, the superoxide can react with neighboring superoxide dismutase (SOD) enzymes that disproportionate superoxide to oxygen and hydrogen peroxide. In fact, NOX enzymes often partner with SODs in signaling processes, whereby SOD converts the cell impermeable superoxide to the diffusible hydrogen peroxide signaling molecule [1–5]. NOX-SOD interactions are also prevalent during infection where the microbial pathogen uses its arsenal of extracellular SODs to combat the oxidative burst of host NOX enzymes [6].

The opportunistic fungal pathogen *Candida albicans* has evolved with a family of three extracellular SOD enzymes (Sod4, Sod5, Sod6) believed to protect the fungus from the attack of host NOX-derived superoxide [7, 8]. We recently reported that these extracellular SODs represent a novel class of Cu-only SOD enzymes that are unique to the fungal kingdom and oomycetes [9, 10]. Much of what is known about fungal Cu-only SODs has emerged from studies on *C*. *albicans* Sod5. Sod5 can react with superoxide at rates limited only by diffusion [9, 10], and can effectively degrade superoxide radicals derived from macrophage and neutrophil NOX enzymes [11, 12].

Curiously *C*. *albicans* Sod5 appears specific to the filamentous form of the fungus [7, 13]. *C*. *albicans* is a polymorphic fungus that can transition from unicellular yeast-like form to pseudo hyphal and true hyphal filamentous states [14, 15]; Sod5 is evidently absent in the yeast-form of *C*. *albicans*. The rationale for selective expression of Sod5 during morphogenesis was not clear, as both yeast and hyphal forms exist in the animal host, are subject to immune surveillance and are essential for virulence [15–17]. Moreover, *SOD5* is induced in filamentous *C*. *albicans* in the absence of any insult from the host [7, 13]. This raises the possibility that filamentous *C*. *albicans* witness a source of superoxide not seen in the yeast-like form.

Certain multicellular fungi are capable of generating superoxide themselves using fungal NOX enzymes as part of signaling during differentiation [18–20]. However, unicellular yeasts were believed to not express NOX, as NOX was characterized as a property of multicellular differentiation [21, 22]. This dogma of no NOX in unicellular fungi was recently challenged by the identification of *Saccharomyces cerevisiae* Yno1, a NOX that localizes to the endoplasmic reticulum and generates intracellular (not extracellular) superoxide [23]. Other than Yno1, there has been no evidence for NOX enzymes in evolutionarily related yeasts including *C*. *albicans*.

Here we provide the first evidence for a NOX enzyme in the opportunistic fungal pathogen *C*. *albicans*. This NOX, known as Fre8, produces a burst of extracellular ROS in filamentous but not yeast-form cells. We demonstrate that Fre8-superoxide serves as substrate for Sod5, providing a rationale for inducing this extracellular SOD during morphogenesis. Strikingly, the ROS from Fre8 is concentrated at the growing tip of *C*. *albicans* hyphae and can promote formation and/or maintenance of elongated hyphae *in vitro* as well as in infected kidneys during a mouse model for disseminated candidiasis. Moreover, Fre8 enhances *C*. *albicans* survival in a rat venous catheter model of *candidiasis*. These studies show that host NOX is not the only source of ROS at the host-pathogen interface; *C*. *albicans* makes its own ROS for hyphal morphogenesis through Fre8.

## Results

### *C*. *albicans* hyphae produce a burst of ROS that serves as substrate for extracellular Sod5

**Fig 1:**
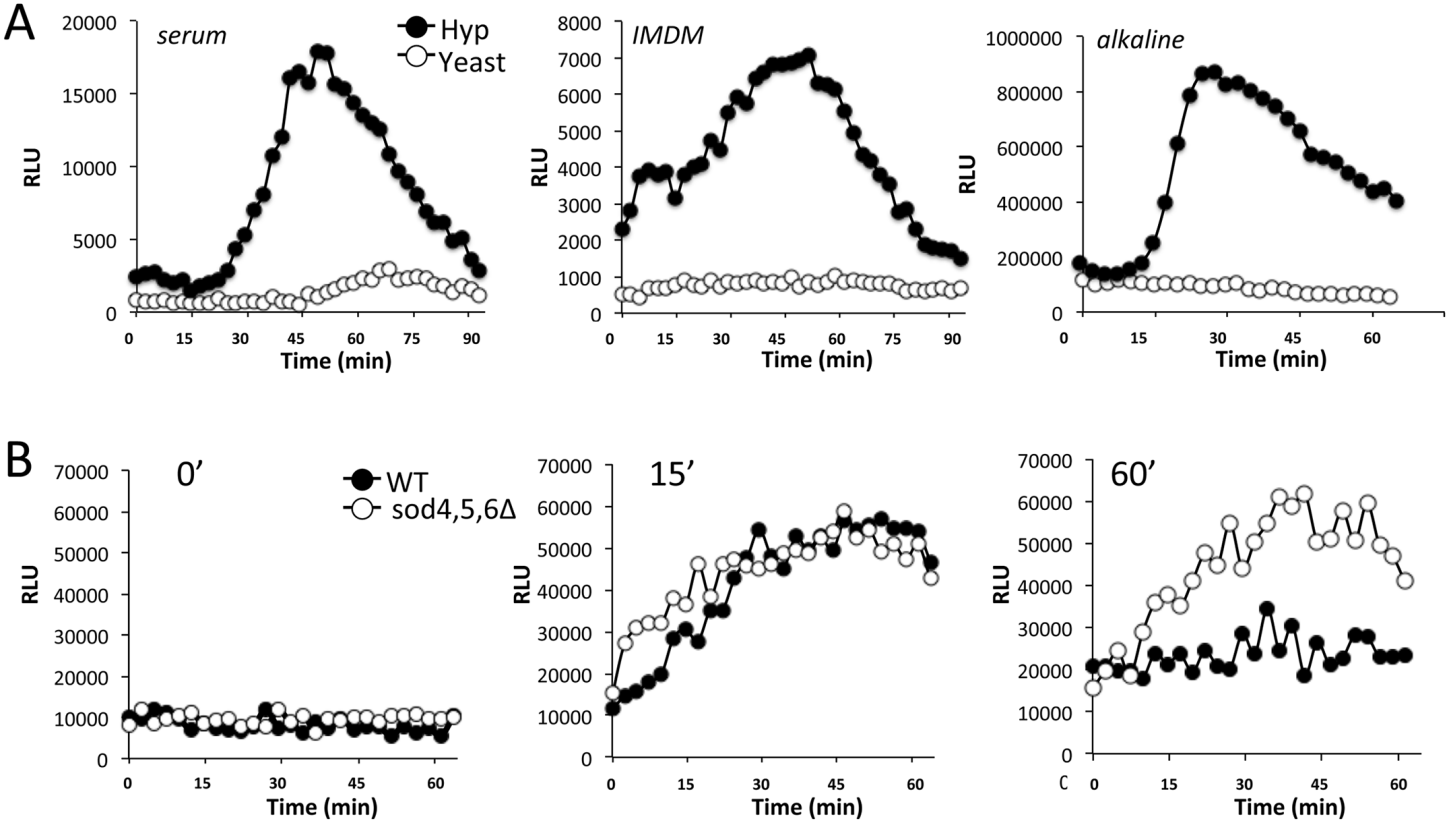
The ROS burst of *C*. *albicans* during morphogenesis. (A) WT strain SC5314 was grown to mid log phase in YPD media at 30°C to obtain the yeast/budding form (“yeast”). Where indicated, cells were induced to form hyphae (“Hyp”) at 37°C for 1 hr with 10% FBS, IMDM or alkaline medium. Both forms of cells were subjected to ROS analysis by luminol chemiluminescence as described in *Materials and Methods*. (B) WT CAIF100 or the isogenic *sod4*Δ/Δ *sod5*Δ/Δ *sod6*Δ/Δ strain were induced to form hyphae in alkaline medium for the indicated times and subjected to ROS measurements by lucigenin chemiluminescence. Results were recorded as relative luminescence units (RLU) as described in *Materials and Methods* and plotted in intervals of whole minutes.

Previously, Schroter et al. demonstrated that *C*. *albicans* produces ROS during the transition from the yeast form to the hyphal state [24]. To probe this fungal ROS, we used luminol, a chemiluminescence probe typically used to measure ROS bursts in macrophages and neutrophils [11, 12, 25, 26]. Consistent with findings by Schroter et al [24], *C*. *albicans* cells induced to form hyphae by serum treatment exhibited a burst in luminol chemiluminesence not seen in yeast-form cells (Fig 1A). We observed that this ROS is not unique to serum stimulation but is also seen when morphogenesis is induced by elevated amino acid concentrations in IMDM medium and alkaline pH conditions (Fig 1A) [14, 27].

**Fig 2:**
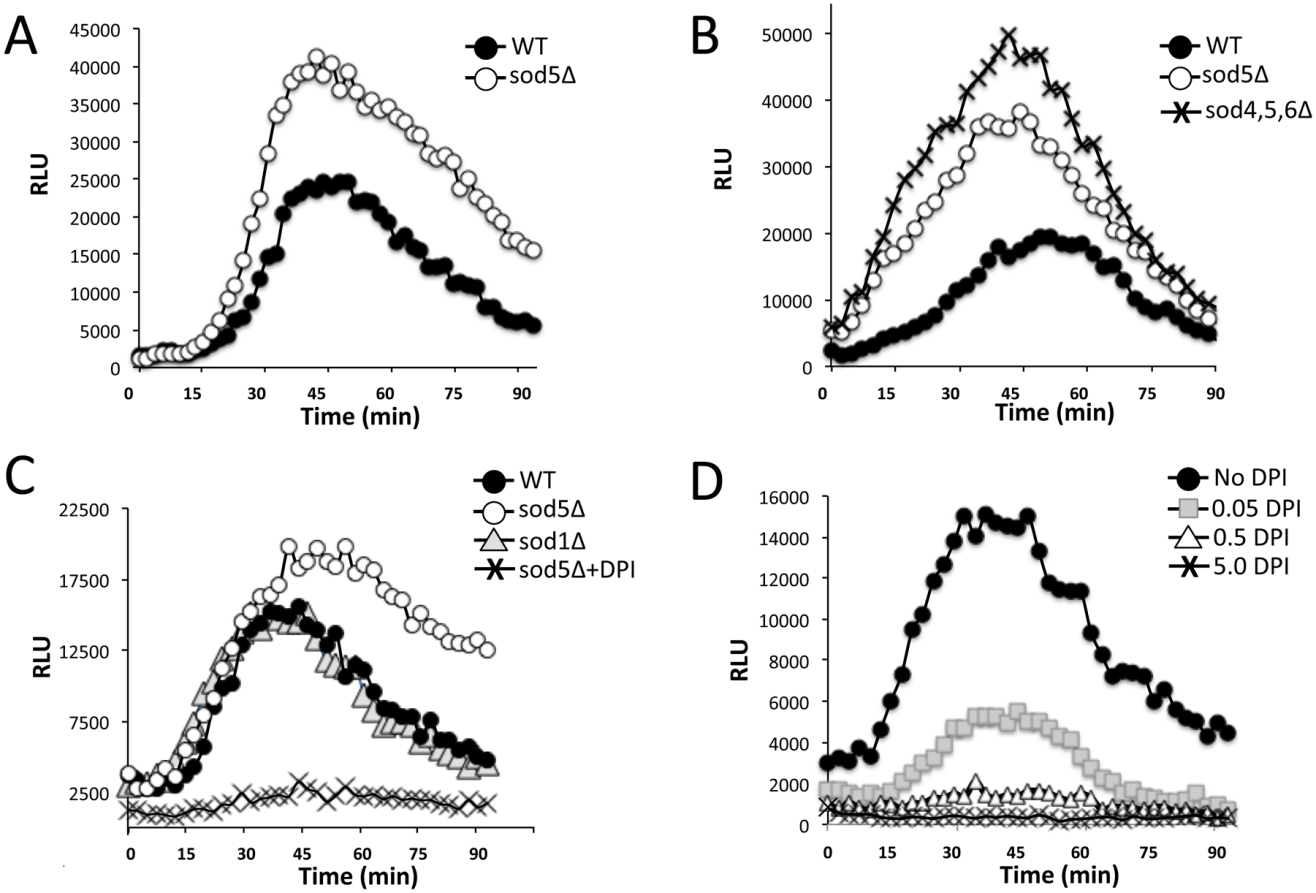
The effect of *sod5*Δ/Δ mutations and NOX enzyme inhibition on the ROS burst of *C*. *albicans*. The indicated *C*. *albicans* strains were induced to form hyphae with either 10% serum (A) or IMDM (B, C, D) and ROS production monitored by luminol as in Fig 1. The indicated strains used are (A) WT SC5314 or the isogeneic *sod5*Δ/Δ cell; (B, C) WT CAIF100 or the isogenic *sod5*Δ/Δ, *sod1Δ/Δ* or *sod4*Δ/Δ *sod5*Δ/Δ *sod6*Δ/Δ strains; (D) WT SC5314. Where indicated, assays were conducted in the presence of the designated concentrations of the NOX inhibitor diphenylene iodonium (DPI) or with 0.5 μM DPI (C).

The luminol probe used in Fig 1A is not expected to penetrate the fungal cell wall, and should therefore only detect extracellular ROS. This notion of extracellular ROS was corroborated using the chemiluminescence probe lucigenin that cannot cross cell membranes, and is specific for superoxide compared to luminol which can detect both superoxide and hydrogen peroxide [25, 28]. As seen in Fig 1B, cells induced to form hyphae exhibited a defined lucigenin signal within 15 min of morphogenesis. However, in WT cells the lucigenin signal often declined at later times points (60 min, Fig 1B), and we tested whether this reflected induction of extracellular SOD enzymes. Indeed the superoxide signal from lucigenin was enhanced in *sod4*Δ/Δ *sod5*Δ/Δ *sod6*Δ/Δ cells lacking all three extracellular SODs (60 min, Fig 1B) and the same was true with luminol chemiluminescence (Fig 2B). Of the three extracellular SODs, deletion of *SOD5* alone was sufficient to enhance ROS during morphogenesis stimulated by serum (Fig 2A) or IMDM (Fig 2B), consistent with the notion that Sod5 is the major extracellular SOD induced during hyphal formation [7]. Compared to effects of *sod5*Δ/Δ mutations, there was no change in luminol ROS in *sod1*Δ/Δ mutants lacking the major intracellular Sod1 (Fig 2C). Together these studies demonstrate that cells undergoing morphogenesis produce a burst of extracellular ROS including superoxide that can serve as substrate for extracellular Sod5.

In multicellular organisms, extracellular ROS is derived from NOX enzymes, although *C*. *albicans* was not previously known to express NOX. To address whether the ROS burst of morphogenesis was derived from a NOX enzyme, we used DPI (diphenylene iodonium), a classical inhibitor of NOX enzymes [29–31]. As seen in Fig 2D, there was a dose response inhibition of the ROS burst of *C*. *albicans* using DPI, with full inhibition at 0.5 μM. DPI also eliminated the enhanced ROS of *sod5*Δ mutants (Fig 2C). These studies suggested that *C*. *albicans* expresses a NOX enzyme for extracellular ROS during morphogenesis. However, since DPI can also inhibit other flavin containing enzymes [32], we applied molecular genetic approaches to examine the source of the ROS burst.

### *C*. *albicans FRE8* encodes a NOX enzyme

In the fungal kingdom, NOX enzymes are part of an expanded family of NADPH oxidoreductases that use electrons from NADPH to either reduce oxygen to superoxide (NOX enzymes) or reduce ferric or cupric metal ions (FRE enzymes) [33]. NOX and FREs are highly similar and it is difficult to predict functionality based on sequence analysis alone [23, 33, 34]. Yeasts are generally thought to only express FRE, not NOX [21, 22] although as mentioned above, this dogma was challenged by identification of *S*. *cerevisiae* Yno1 as an endoplasmic reticulum NOX [23]. In *C*. *albicans*, there are at least 17 genes annotated as FREs [35, 36], three of which are known cupric or ferric reductases (Fre1, Fre7, Fre10 (S1 Fig, [37–39]); the remainder have uncharacterized functions. By qRT-PCR, we identified a number that are induced during early stages of hyphal morphogenesis coincident with the ROS burst (S1 Fig). The *C*. *albicans* orthologue to *S*. *cerevisiaeYNO1* [18] was not among the FREs induced with morphogenesis (S1 Fig). The most highly induced hyphal specific gene was *FRE8*, also known as *CFL11* or *C*. *albicans* CR_06670W, orf19.701 (S1 Fig). *FRE8* was additionally reported as the most abundantly induced FRE during *C*. *albicans* invasion of the kidney [40]. We chose to focus on *FRE8* as a potential NOX enzyme.

**Fig 3:**
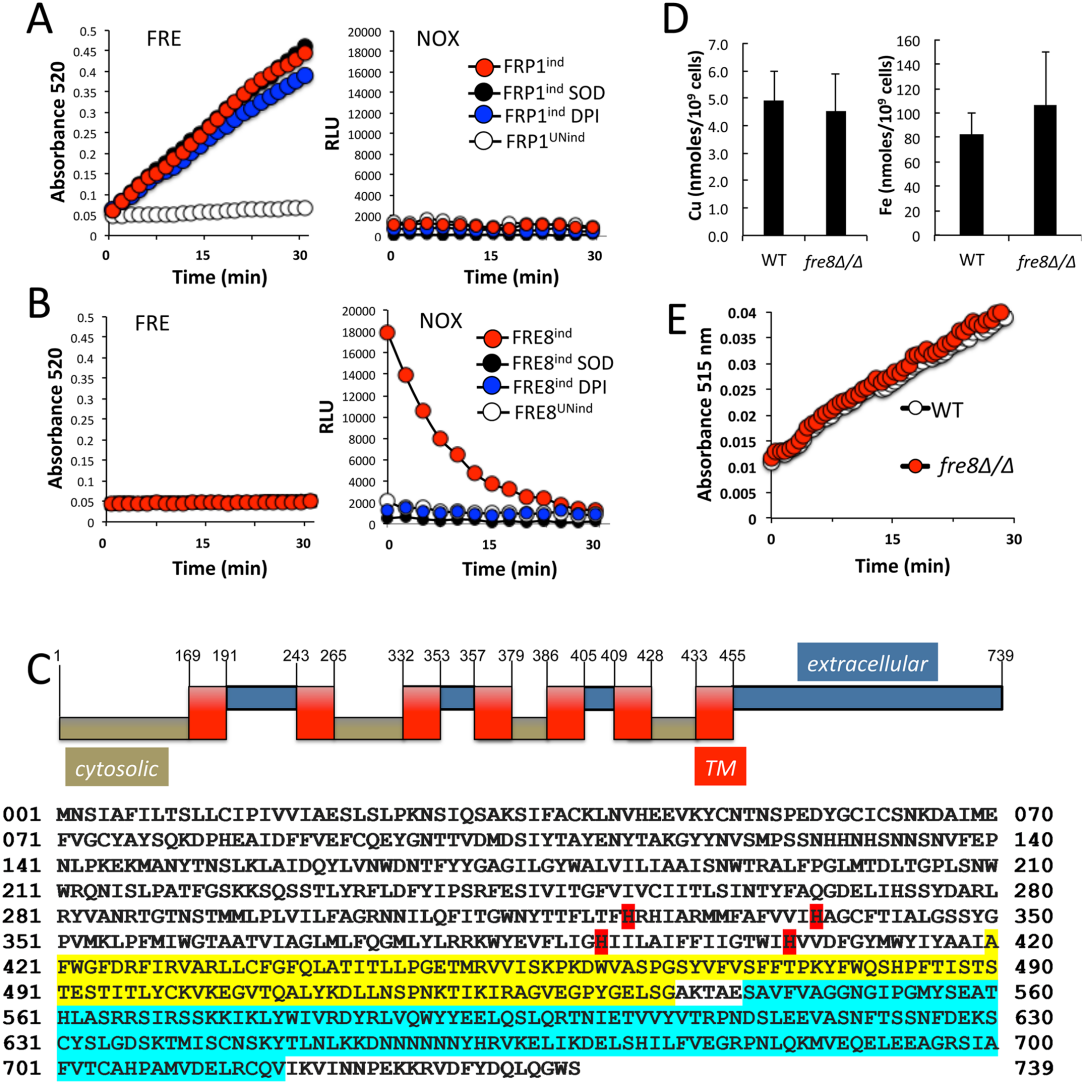
*C*. *albicans FRE8* as a candidate NOX enzyme. (A, B) *P*. *pastoris* strains expressing recombinant *C*. *albicans FRP1* (A) or *C*. *albicans FRE8* (B) under control of the *AOX2* promoter were cultured with either methanol to induce gene expression (“ind”) or with glycerol (“UNind”) to prevent expression of recombinant *FRP1* or *FRE8*. Samples were analyzed for ferric reductase (“FRE”) activity (A, B left) or for NOX-like activity (“NOX”) through ROS production by luminol chemiluminescence (A,B right) as described in *Materials and Methods*. Compared to *C*. *albicans*, the luminol substrate appears rapidly depleted in *P*. *pastoris* expressing high levels of *FRE8*. Where indicated, assays were supplemented with 0.1 unit bovine Cu/Zn SOD1 to remove extracellular superoxide or 50 nM DPI to inhibit NOX activity. Although DPI is predicted to inactivate other flavin requiring enzymes [32], this dose of DPI does not inhibit the ferric reductase activity of recombinant Frp1. (C) *TOP*- predicted transmembrane domain of *C*. *albicans* Fre8 based on TMHMM hydropathy plot analysis where brown and blue bars are predicted cytosolic and extracellular/luminal domains respectively and red are transmembrane (TM). *BOTTOM*–amino acid sequence of *C*. *albicans* Fre8 showing predicted histidine ligands for heme (red), FAD (yellow) and NAD (aqua) binding as determined by InterPro and PFAM databases. (D, E) WT SC5314 and isogenic *fre8*Δ/Δ *C*. *albicans* cells induced to form hyphae for 1 hour were analyzed for copper and iron accumulation by ICP-MS (D) or for ferric reductase activity (E) as described in *Materials and Methods*. ICP-MS results represent the averages of three biological replicates where error bar equals standard deviation. Based on T-test there were no significant differences with either copper (P = 0.73) or iron (P = 0.47) accumulation.

Recombinant versions of codon optimized *FRE8* were expressed in *Pichia pastoris* under control of the methanol inducible *AOX2* promoter. An identical procedure has been used to express and analyze activity of mammalian NOX enzyme complexes [41]. In parallel, we expressed a second unknown member of the FRE family, namely *FRP1*, that is only moderately induced by hyphal stimulation (S1 Fig). *P*. *pastoris* cells expressing recombinant *FRE8* and *FRP1* were assessed for ferric reductase activity and ROS production. As seen in Fig 3A left, *Pichia* cells expressing *FRP1* exhibited clear ferric reductase activity when *FRP1* expression was induced with methanol. These cells however, exhibited no ROS that could be detected by luminol (Fig 3A right), indicating that *FRP1* encodes a metalloreductase, and not a NOX enzyme. The opposite profile was obtained with *Pichia* cells expressing *FRE8*. These cells exhibited no methanol-inducible ferric reductase activity (Fig 3B left), but produced a strong methanol-inducible ROS signal (Fig 3B right). This ROS was eliminated by addition of 0.5 μM of the NOX inhibitor DPI or by addition of exogenous SOD enzyme (bovine SOD1). By contrast the ferric reductase activity of recombinant Frp1 was not altered by exogenous SOD or by DPI (Fig 3A left). These results indicated that *FRP1* encodes a metalloreductase while *FRE8* encodes a NOX.

**Fig 4:**
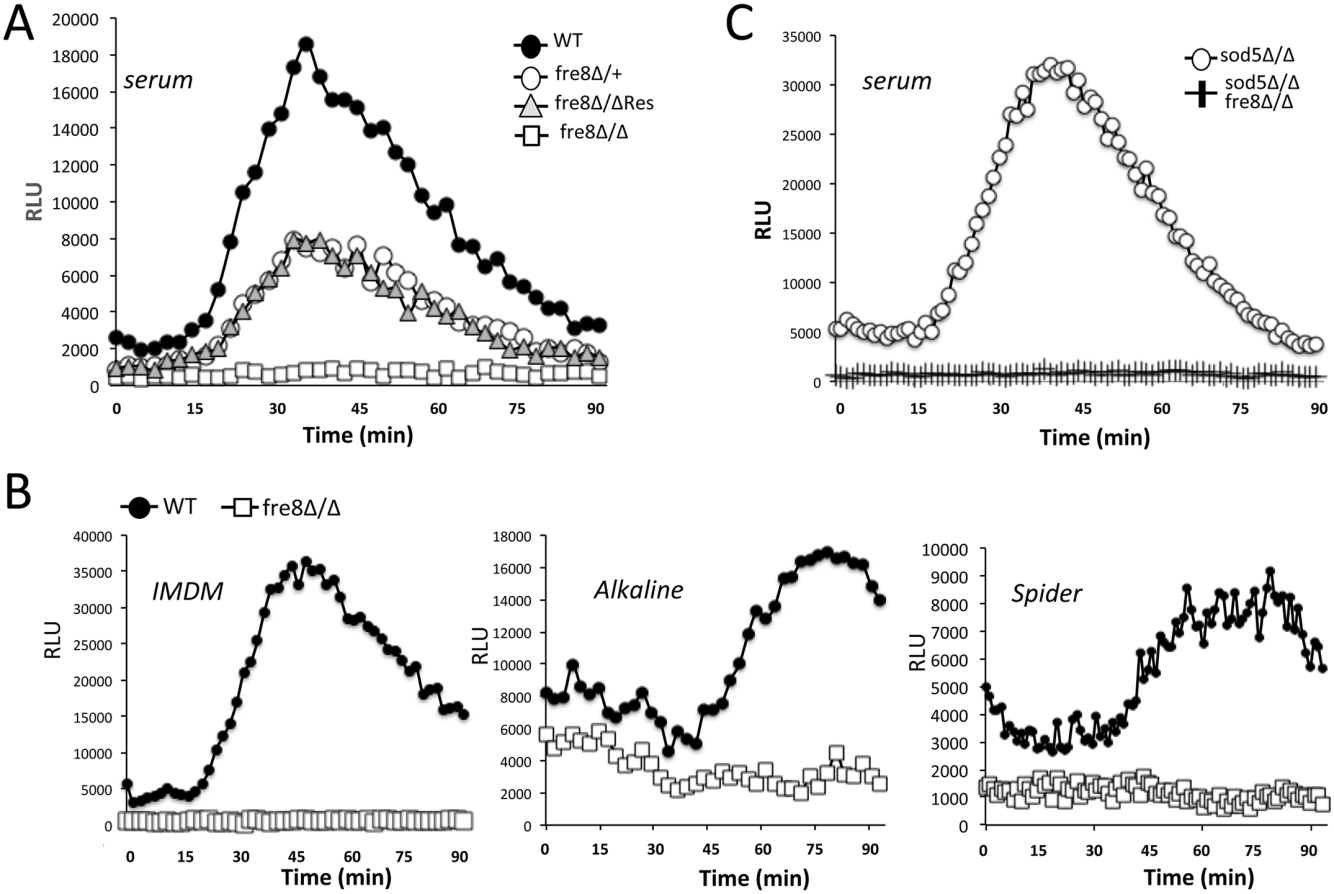
The ROS burst of *C*. *albicans* morphogenesis is eliminated by *fre8*Δ/Δ mutations. The indicated strains were induced to form hyphae by either 10% serum (A,C) or by IMDM, alkaline or Spider medium (B) as described in *Materials and Methods* and ROS formation by luminol was monitored as in Fig 1. The *fre8* heterozygous “fre8Δ/+” or *fre8*Δ/Δ complemented with a single copy of *FRE8* (“fre8Δ/ΔRes”) exhibit haploinsufficiency with regard to ROS formation. All strains were in the background of SC5314.

Fre8 contains all the features predicted for a member of the NADPH family of oxidoreductases, including seven transmembrane domains and sequences for binding heme, FAD and NADPH (Fig 3C). To assess the function of Fre8 *in vivo*, we deleted both copies of genomic *FRE8* in *C*. *albicans* and tested effects on metals and ROS formation. We observed no impact of *fre8*Δ/Δ mutations on accumulation of copper or iron (Fig 3D) or on whole cell ferric reductase activity (Fig 3E). However, the *fre8*Δ/Δ strain was completely defective in generating ROS, and this result held true regardless of the stimuli for morphogenesis including serum (Fig 4A), alkaline medium, IMDM and spider medium (Fig 4B). The heterozygous *fre8*Δ/+ strain retaining a single genomic copy of *FRE8* exhibited roughly a 50% reduction in ROS generation and the same haploinsufficiency was seen with the *fre8*Δ/Δ strain complemented with a single copy of *FRE8* (Fig 4A). As previously mentioned (Fig 2), the ROS burst of hyphal cells is enhanced in a *sod5*Δ/Δ mutant, and as shown in Fig 4C, this elevated ROS is Fre8-mediated, as no ROS is detected in a double *sod5*Δ/Δ *fre8*Δ/Δ mutant. The ROS burst emitted by *C*. *albicans* during the morphogenic switch is clearly Fre8-dependent.

**Fig 5:**
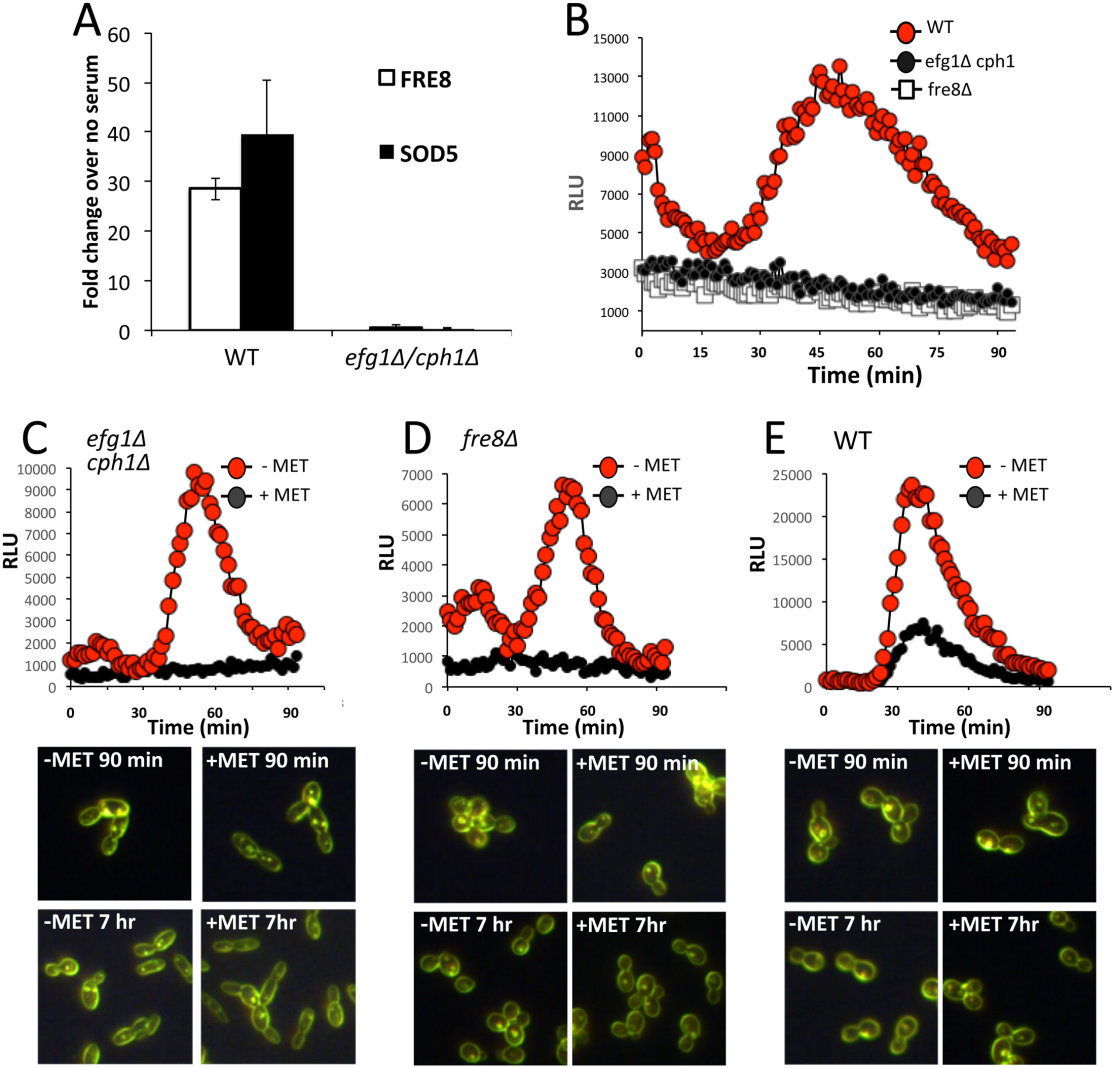
*C*. *albicans* ROS production as a function of *FRE8* expression. (A,B) The indicated strains were induced to form hyphae with 10% FBS for 1 hour and were tested for (A) *FRE8* and *SOD5* mRNA by qRT-PCR or (B) ROS formation by luminol chemiluminescence as in Fig 1. (A) mRNA levels were compared to that of yeast-form cells prior to serum addition. Results represent the averages of 4 samples over two independent trials, error bars equal standard error. (C-E) The indicated strains engineered to express *FRE8* from the *MET3* promoter were grown to mid log phase in SC medium either containing methionine (+MET) to repress *FRE8*, or lacking methionine (-MET) to de-repress *FRE8* expression for either 1 hour (Top) or the indicated time points (Bottom). Cells were examined for (Top) ROS production by luminol chemiluminescence and (Bottom) dark field microscopy for cell morphology at 40X magnification. Images were taken of cells following the indicated times of de-repressing *FRE8* expression. All strains are in the background of SC5314.

We observed that *FRE8* mRNA is induced within 1 hour of serum treatment (Fig 5A), congruent with the ROS burst (Fig 1A) and the induction of *SOD5* (Fig 5A). In *C*. *albicans*, morphogenesis involves complex signaling pathways that converge on the Efg1 and Cph1 transcription factors, and *efg1*Δ/Δ *cph1*Δ/Δ null cells are incapable of forming hyphae [14, 42]. We observed that *efg1*Δ/Δ *cph1*Δ/Δ mutations block induction of *FRE8* and *SOD5* by serum (Fig 5A) and accordingly, the ROS burst is also eliminated (Fig 5B). *SOD5* has previously been shown to fall under control of Efg1 [13]. *SOD5* and *FRE8* were not identified by ChIP as direct targets of Efg1 or Cph1 [43] [44]; both are subject to chromatin remodeling control by Hir1 that works in concert with Efg1 to control genes for morphogenesis [45].

Is hyphal formation required for ROS production? We uncoupled *FRE8* expression from morphogenesis through ectopic expression of *FRE8* under control of the repressible *MET3* promoter in *cph1*Δ/Δ *efg1*Δ/Δ cells, *fre8*Δ/Δ cells and WT *C*. *albicans*. Strains were grown under conditions favoring yeast-only growth, and then *FRE8* expression was de-repressed by removal of methionine. As seen in Fig 5C–5E there was a burst of ROS from all cells in which *FRE8* expression was de-repressed. Importantly, these cells remained in the budding yeast-form, with no evidence of hyphal forms or germ tubes (Fig 5C–5E bottom). Thus the ROS burst is not dependent on morphogenesis, but rather the expression of *FRE8*, regardless of the morphogenic state. This study also demonstrated that Fre8-ROS is not sufficient to induce hyphal formation.

**Fig 6:**
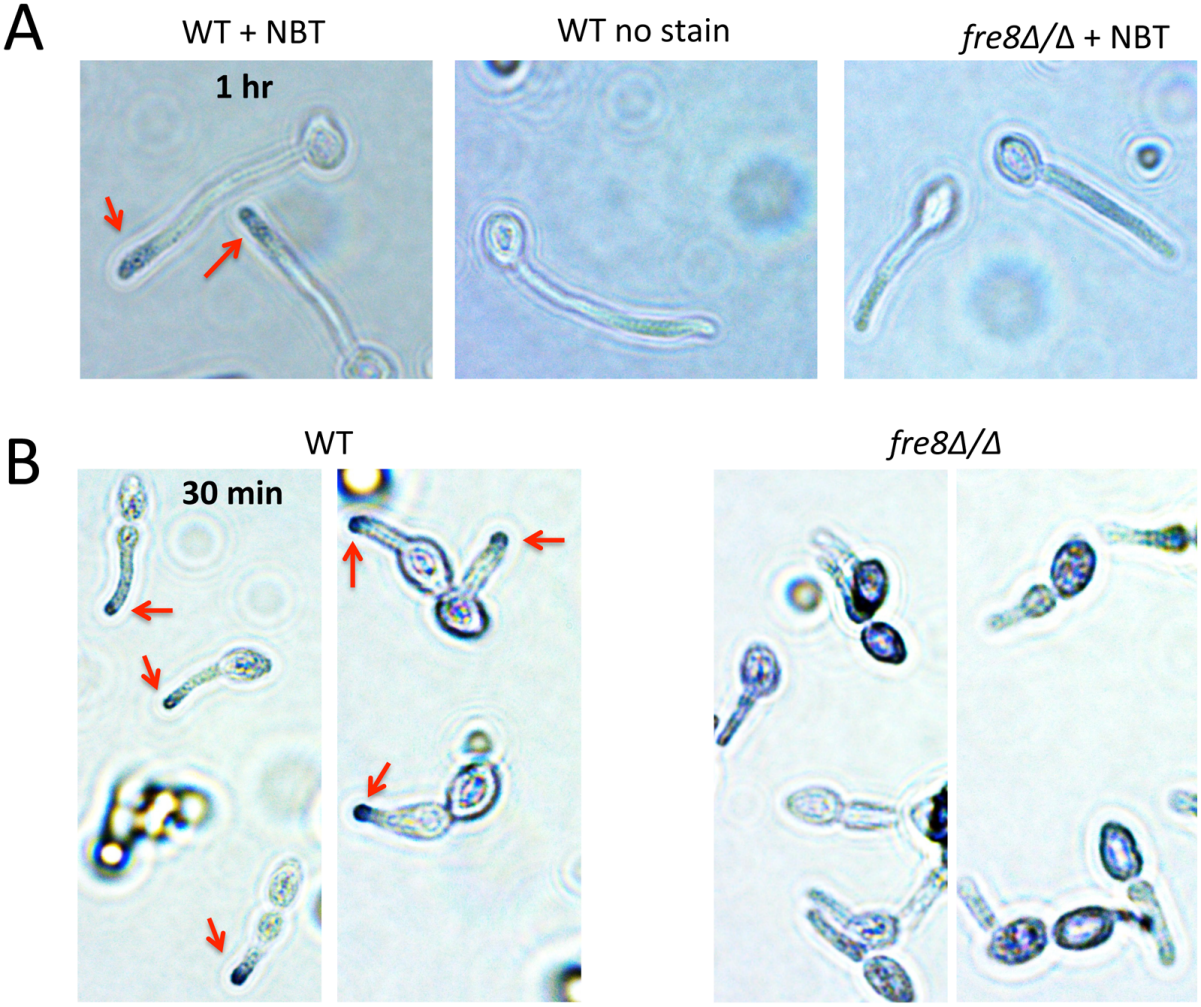
Evidence for ROS production by Fre8 at the growing tip of *C*. *albicans* hyphae. The designated cells were induced to form hyphae in 10% serum as in Fig 1A at a density of 4 x 10^6^ cells/ml for either 60 (A) or 30 minutes (B). Cells were stained with nitroblue tetrazolium (NBT) as described in *Materials and Methods* and subjected to light microscopy at a magnification of 100X.

We next examined localization of Fre8-dependent ROS using nitrobluetetrazolium (NBT) which has been used to localize NOX superoxide in multicellular fungi [46, 47]. NBT is reduced by superoxide, forming a purple formazan precipitate. As seen in Fig 6, intense NBT staining was observed at the tip of elongating germ tubes in WT cells but not *fre8*Δ/Δ mutants (Fig 6A). This staining was discernable within 30 min of stimulating morphogenesis (Fig 6B). Thus, Fre8-dependent ROS is specific to the growing tip of the polarized cell.

### A role for Fre8 in hyphal development *in vitro*

**Fig 7:**
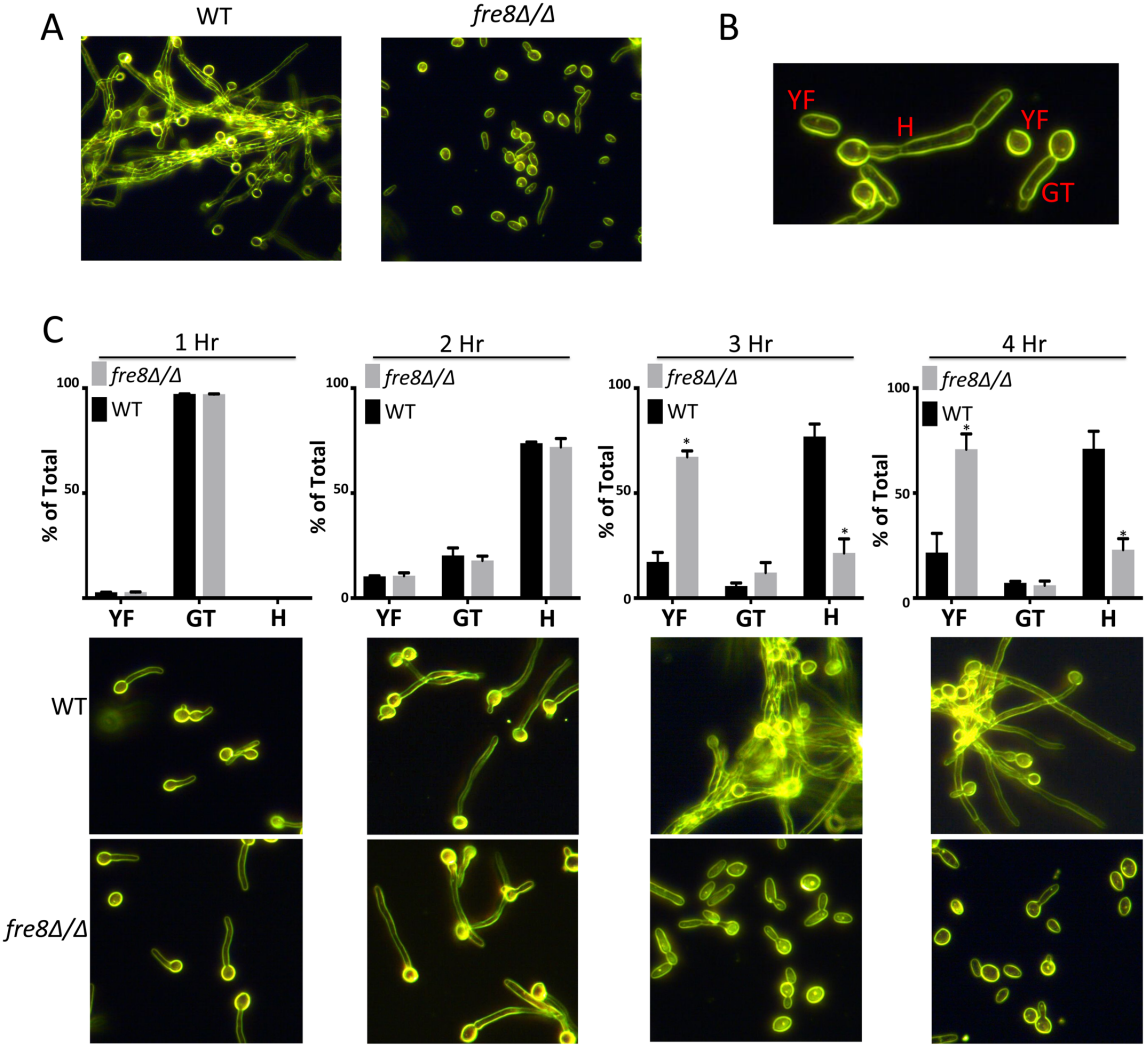
Hyphal defect of *fre8*Δ/Δ cells. (A) WT SC5314 or the isogeneic *fre8*Δ/Δ strain were seeded at 6 x 10^7^ cells/ml and induced to form hyphae at 37°C with 10% serum. Following 4 hours (A) or the indicated time points (C), cells were photographed by dark field microscopy at 40X magnification. Shown are accurate representatives of 4–8 images. (B) Examples of the different morphological forms identified and quantified in the graphs of C, top. “Y”, yeast-form including rounded, oval or short oblong morphologies; GF, germ-tubes; H, hyphal. (C top) Roughly 350 cells for each time point were counted over two experimental trials and were classified according to morphological shapes defined in part B. The differences in yeast-form and hyphal cells in *fre8*Δ/Δ versus WT SC5314 cells is statistically significant (*p<0.05) by two tailed t-test.

**Fig 8:**
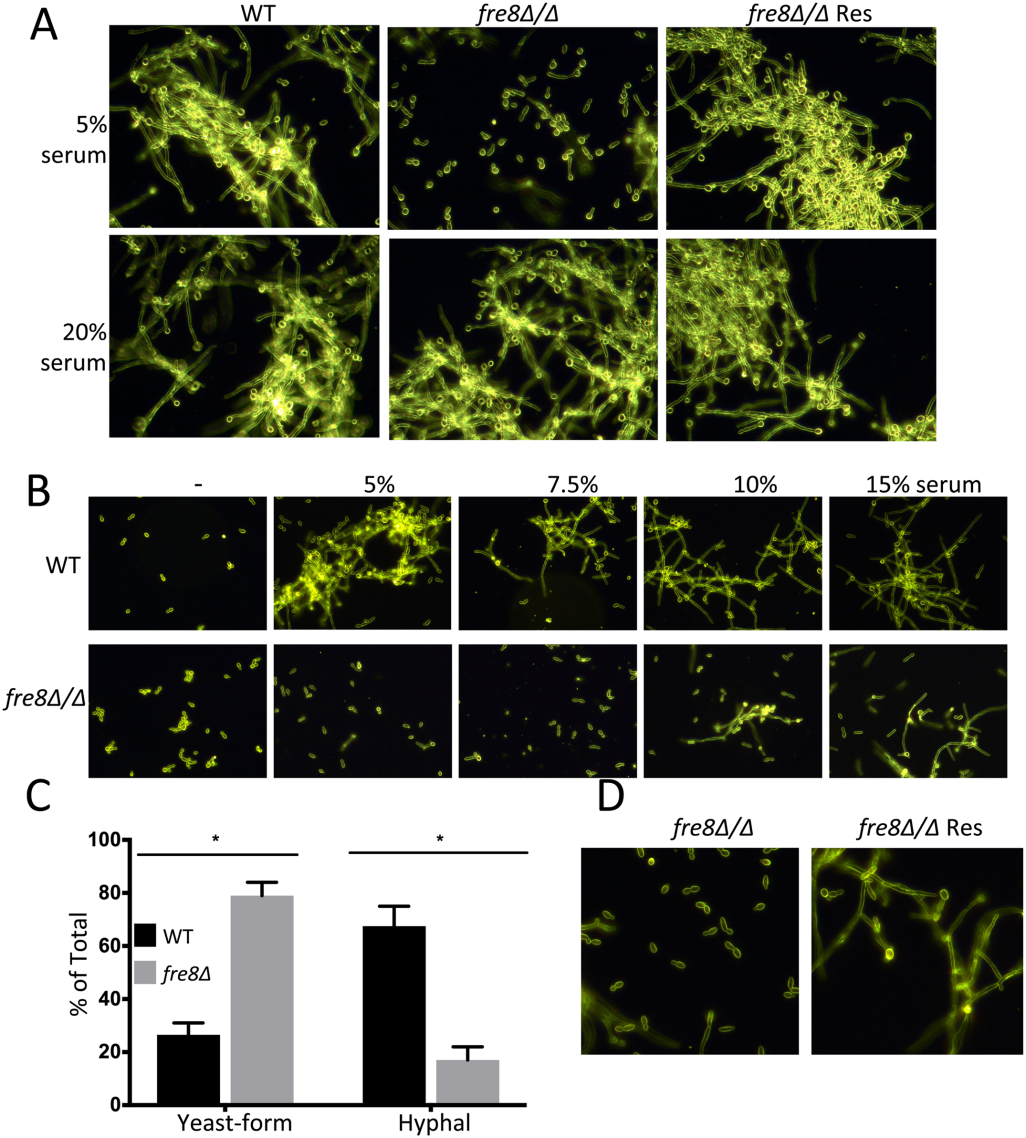
Impact of serum on morphogenesis involving Fre8. WT, *fre8*Δ/Δ and the *FRE8* re-integrant “*fre8*Δ/Δ *Res*” were seeded at either 6 x 10^7^ cells/ml (A) or 4 x 10^6^ cells/ml (B-D) and induced to form hyphae at 37°C or 34°C respectively with the indicated levels of serum (A,B) or with 7.5% serum (C,D). Following 4 hours, cells were photographed (A,B,D) and enumerated (C) as in Fig 7. (C) 300 to 460 WT and *fre8*Δ/Δ cells over two experimental trials were classified into yeast-form and hyphae as in Fig 7C. The difference between WT and *fre8*Δ/Δ cells is statistically significant as determined by two tailed t-test, *p = ≤0.03.

Why would *C*. *albicans* produce ROS during hyphal formation? We considered whether this ROS might act as a signal to modulate morphogenesis, as has been shown for ROS modulating development in differentiating fungi [18–20]. Although no detectable change in morphology could be noted in the NBT experiment of Fig 6, these experiments were conducted at early time points when cells were initially forming germ tubes. However, at later time points when cell formed elongated hyphae, a *fre8*Δ/Δ deficiency could be observed (Fig 7A). This *fre8*Δ/Δ defect was complemented in the *FRE8* re-integrant (Fig 8A top) and appeared completely specific to later stages of hyphal development. After 3–4 hours when WT cells were assembling into elongated hyphae, *fre8*Δ/Δ cells accumulated abundant yeast-forms (Fig 7C).

The experiments of Fig 7 employed cultures seeded at 6 x 10^7^ cells/ml, a relatively high density where quorum sensing is prominent [48]. However, quorum sensing cannot explain the *fre8*Δ/Δ defect, as these mutants showed no increased sensitivity to the quorum sensing molecule farnesol (S2 Fig parts A,B), and high density cultures of *fre8*Δ/Δ cells exhibited WT-like quorum sensing properties [48] (S2 Fig part C and legend). Most importantly, the *fre8*Δ/Δ defect in hyphal development can also be seen in low density cultures not subject to quorum sensing, e.g., when 4 x 10^6^ cells/ml are stimulated with serum at 34°C (Fig 8B–8D). It is important to note that the requirement for Fre8 in hyphal formation is not absolute, and can be bypassed by potent stimuli for morphogenesis, e.g., high levels of serum (Fig 8A bottom) or when low density cells are shifted to temperatures ≥37°C (S2 Fig Part C). We conclude that Fre8 can act as a modifier of hyphal development, but is not unconditionally essential for the process.

**Fig 9:**
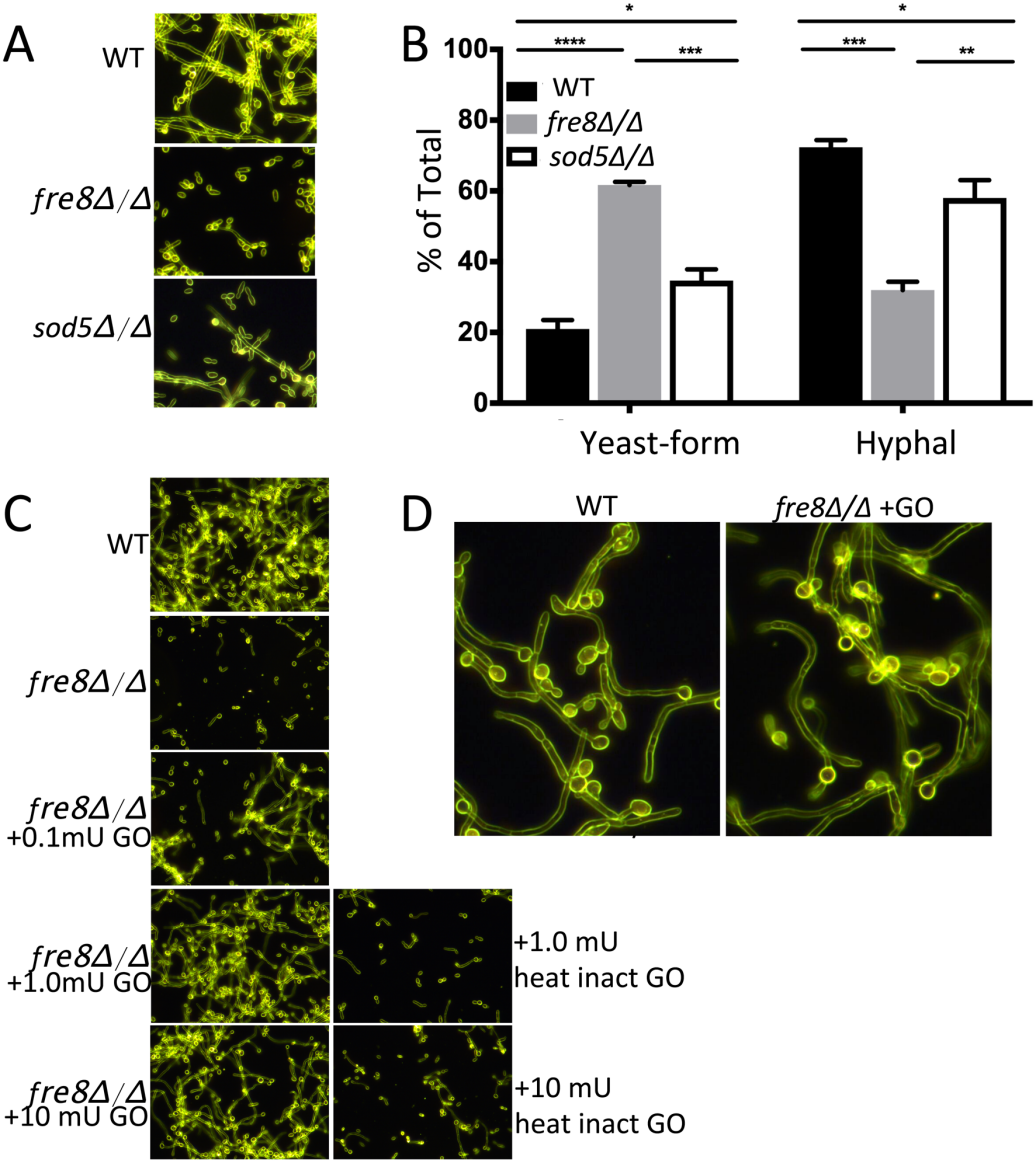
Evidence for a role for H_2_O_2_ in Fre8 control of morphogenesis. The indicated strains were seeded at 6 x 10^7^ cells/ml and induced to form hyphae for 4 hours with 5% serum. (A,B) Shown are comparisons of WT, *fre8*Δ/Δ and *sod5*Δ/Δ cells where photographs (A) are representative of 15 images over three experimental trials and quantification (B) involved classification of 250–400 cells for each of three trials. The comparisons are all statistically significant as determined by one-way ANOVA with Tukey post-test, ****p<0.0001, ***p≤0.0005, **p = 0.004, *p≤0.049. C) The effects of exogenous glucose oxidase (GO) on hyphal formation of *fre8*Δ/Δ cells. Shown are amounts GO added to a 1 ml culture where 1 mU GO catalyzes the production of 1 nmole H_2_O_2_/min. Where indicated, GO was subject to heat inactivation by boiling for 10 min prior to addition to cultures. Results are representative of 6–10 images over two experimental trials. D) Enlargement showing WT-like hyphal development in *fre8*Δ/Δ cells treated with 1 mU GO.

Since Sod5 reacts with Fre8 superoxide to produce H_2_O_2_, we tested whether *sod5*Δ/Δ mutations likewise affect hyphal morphogenesis. As seen in Fig 9A and 9B, *sod5*Δ/Δ mutants exhibited a defect in hyphal development that was less pronounced than that of *fre8*Δ/Δ cells. Such an intermediate effect could be expected since Fre8 superoxide may also be converted to H_2_O_2_ through spontaneous disproportionation [49] or through extracellular Sod4 and Sod6. Even so, the parallel trends seen with *sod5* and *fre8* mutations would imply that H_2_O_2_ (and not superoxide) underlies the Fre8-defect. To more definitely test this, we addressed whether exogenous H_2_O_2_ could bypass the requirement for Fre8 in hyphal development. Previous studies have shown that mM concentrations of H_2_O_2_ can induce hyperpolarized buds in *C*. *albicans* [50, 51], or pseudohyphae [52, 53], neither of which resemble true *C*. *albicans* hyphae. Rather than using a single bolus of H_2_O_2_ as was previously done, we used glucose oxidase (GO) to continuously generate exogenous H_2_O_2_. As seen in Fig 9C, as little as 0.1 mU of GO (generates 100 pmoles H_2_O_2_/min) was able to restore hyphal development to *fre8*Δ/Δ cells while heat-inactivated GO was without effect. It is important to note that the hyphae formed with GO treated *fre8*Δ/Δ cells were indistinguishable from that of WT cells (Fig 9D), unlike the elongated buds and pseudohyphae reported for *C*. *albicans* treated with mM H_2_O_2_ [50–53]. Together, our *in vitro* studies of Figs 7–9 support a model in which the H_2_O_2_ produced by Fre8 can act as a modifier of hyphal morphogenesis.

### The role of Fre8 in rodent models of candidiasis

**Fig 10:**
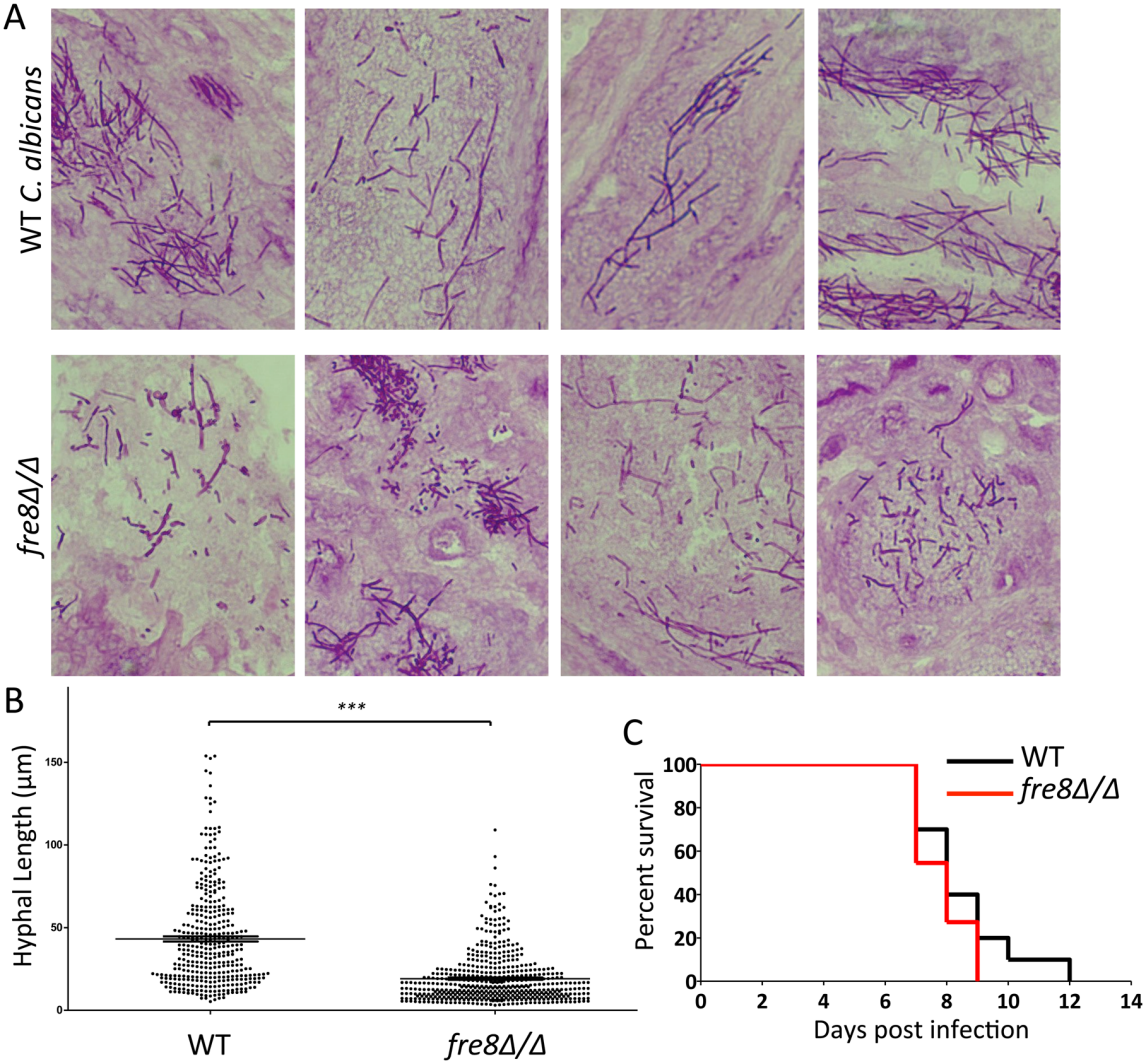
Effect of *fre8*Δ/Δ mutations on fungal invasion of the kidney and virulence in a mouse model of disseminated candidiasis. Mice were infected with either WT SC5314 or the isogenic *fre8*Δ/Δ mutant by the lateral tail vein injection as described in *Materials and Methods*. (A) Following 7 days of infection, kidneys were harvested from surviving mice and analyzed for fungal morphology by PAS staining as in *Materials and Methods*. Shown are the individual kidney sections from 4 independent mice for each group. (B) Quantification of the hyphal length from WT versus *fre8*Δ/Δ fungi invading the kidney. Results represent 375 and 503 fungal cells from 6 WT-infected and 4 *fre8*Δ/Δ-infected mice, respectively. The short oblong morphological forms that were prevalent with *fre8*Δ/Δ cells were included in this analysis, but not the occasional rounded yeast-forms. The difference in hyphal length between WT and *fre8*Δ/Δ cells is statistically significant as determined by t-test, ***p<0.0001. (C) Survival curves of infected mice including 10 mice from each group. There was no statistical difference between mice infected with WT *C*. *albicans* versus the *fre8*Δ/Δ mutant as determined by the log-rank (Mantel-Cox) test.

It was important to examine the impact of Fre8 *in vivo*, as the animal host is the only natural environment for *C*. *albicans*. One model examined was the mouse model for disseminated candidiasis where kidney is the target organ. In late stages of infection, kidneys were harvested from mice infected with WT versus *fre8*Δ/Δ strains and subjected to histological analysis of invading fungi by PAS staining. As seen in Fig 10A top, WT *C*. *albicans* predominantly showed elongated hyphal filaments in the infected kidney. By comparison, the *fre8*Δ/Δ mutant from 4 independent mice produced a mixture of morphological forms with a much larger proportion of shorter filaments or yeast form cells in *fre8*Δ/Δ mutants compared to WT *C*. *albicans* (Fig 10A and 10B). These findings are similar to *fre8*Δ/Δ effects on morphology *in vitro* (Figs 7–9). Yet in spite of the changes in morphology observed *in vivo*, there was no overall impact on pathogenesis with *fre8*Δ/Δ mutants. *fre8*Δ/Δ mutants showed no deficiency in virulence (Fig 10C), and markers of host inflammation [54, 55] were similar between SC5314 and *fre8*Δ/Δ infected kidneys (S3 Fig part A). Colony forming units (CFUs) in the kidney 48 hours post infection were modestly (2–3 fold) lower in *fre8*Δ/Δ mutations relative to WT (S3 Fig part B). All morphological forms are thought to contribute to infection and invasion [14], and our data with *fre8*Δ/Δ mutants supports this view.

**Fig 11:**
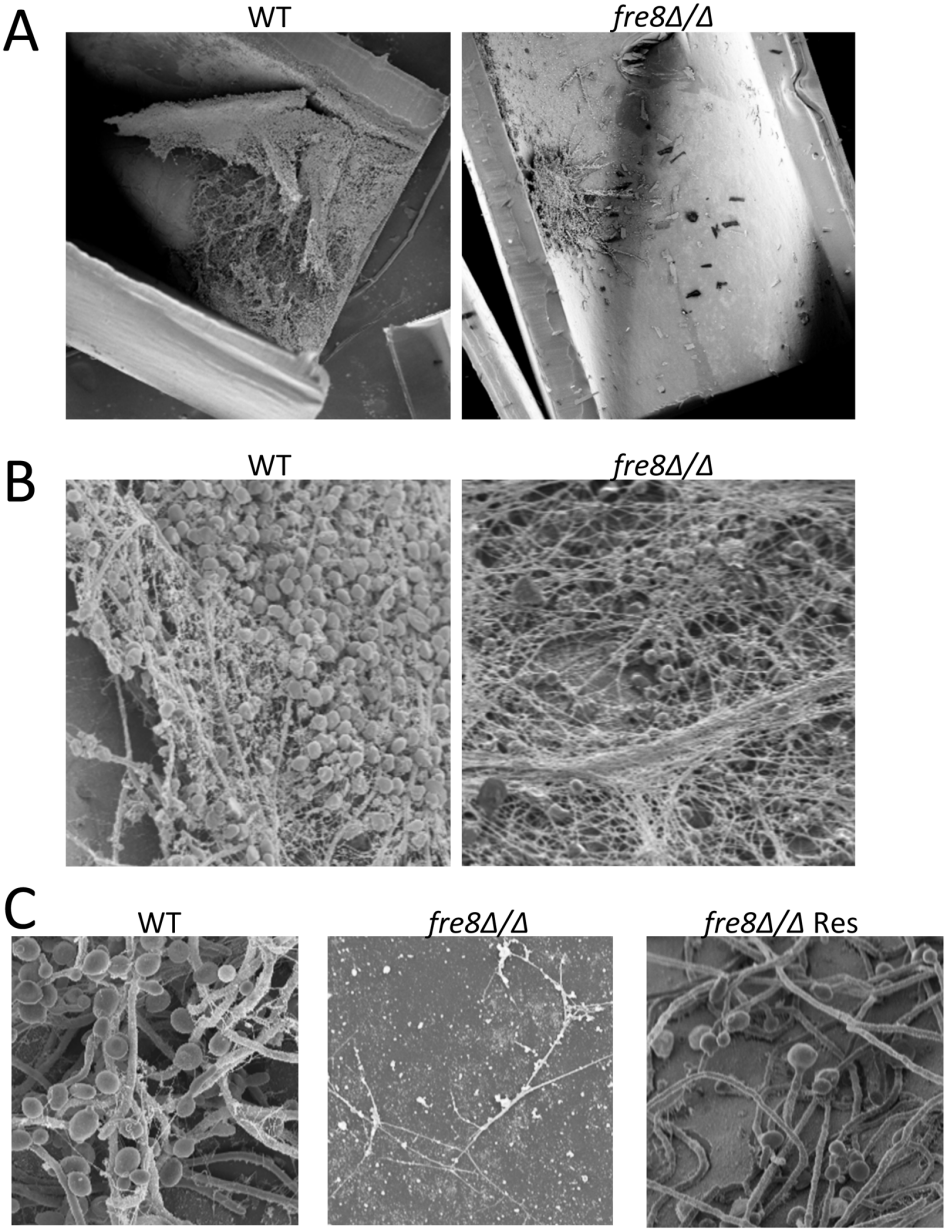
Effect of *fre8*Δ/Δ mutations in a rat model of biofilm formation. SC5314 or the isogenic *fre8*Δ/Δ strain or the complemented *fre8*Δ/Δ harboring a single *FRE8* allele (“*fre8*Δ/Δ Res”) were tested for biofilm formation in the rat venous catheter model as described in *Materials and Methods*. Results in A-C are from three independent experimental trials. SEM images shown were taken at 80X (A), 1000X (B) and 2000X (C) magnification.

We additionally tested the *fre8*Δ/Δ mutant in a rodent model of catheter biofilms. *C*. *albicans* is capable of forming surface adherent aggregates of biofilms on either biological surfaces (e.g., epithelial cells) or on medical implant devices, and such dense fungal communities are highly tolerant to antifungals [56]. One of the most common clinical biofilm infections involves venous catheter implants and a rodent vascular catheter model faithfully recapitulates the human disease [56]. In this model, WT SC5314 *C*. *albicans* forms robust biofilms within 24 hours of injection into the animal catheter (Fig 11A–11C). Over three independent trials, *fre8*Δ/Δ cells exhibited deficiencies whereby the biofilms were either sparse in number (Fig 11A) or undetected (Fig 11C), and when present, biofilms were often attenuated with few elongated hyphae (Fig 11B). This defect was partially reversed with the *Fre8* re-integrant (Fig 11C), consistent with the haploinsufficiency and partial restoration of ROS formation *in vitro* (Fig 4A).

**Fig 12:**
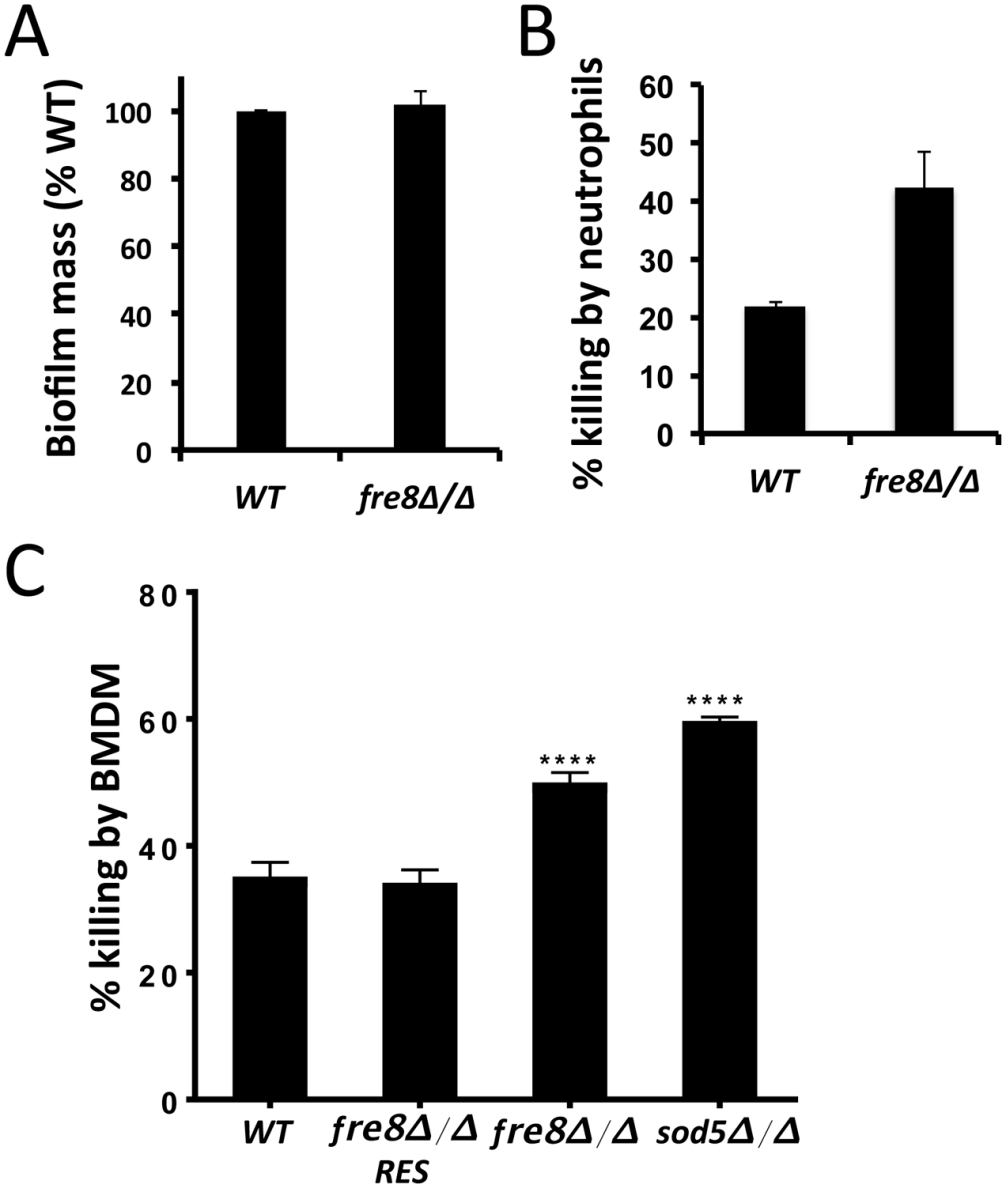
Susceptibility of *fre8*Δ/Δ cells to killing by neutrophils and macrophages. (A) SC5314 WT or the isogenic *fre8*Δ/Δ mutant biofilms were grown for 24 hours in 96 well plates and the relative fungal burden was estimated using the XTT assay as described in *Materials and Methods*. Results were normalized to SC5314 biofilm allowing for averaging of 3 independent experiments. (B) 24 hour SC5314 or *fre8*Δ/Δ biofilms were incubated with or without human neutrophils (E:T ratio 1:2) for 4 hours and an XTT metabolic assay was used to estimate fungal burden. No biofilm controls were included to estimate neutrophil contribution to XTT assays. Neutrophil contributions were subtracted from biofilm XTT assays to calculate fungal inhibition. Results represent the averages of 3 independent experimental trials. The increase in inhibition of *fre8*Δ/Δ cells by neutrophils was statistically significant (P ≤ 0.04 by T-test). (C) WT SC5314 or the isogenic *fre8*Δ/Δ, *sod5*Δ/Δ or the *FRE8* complemented *fre8*Δ/Δ (*fre8*Δ/Δ Res) were tested for killing by BMDM as described in *Materials and Methods*. Results represent the averages of 9 samples over two experimental trials. The increased killing of *sod5*Δ/Δ and *fre8*Δ/Δ cells compared to WT or to the *fre8*Δ/Δ Res was statistically significant ****p<0.0001 by one-way ANOVA with Tukey post-test. There was no statistically significant difference between WT and *fre8*Δ/Δ Res, while the increased killing in *sod5*Δ/Δ compared to *fre8*Δ/Δ was significant (p = 0.004) by one-way ANOVA with Tukey post-test.

The *fre8*Δ/Δ defect in biofilms *in vivo* may very well reflect changes in morphogenesis similar to what we observed *in vitro* (Figs 7–9). Yet host factors may also contribute. Neutrophils represent the primary leukocytes of *Candida* biofilms in catheters [57] and we tested whether *fre8*Δ/Δ cells were more sensitive to neutrophil killing. As seen in Fig 12A, the total mass of *C*. *albicans* biofilms *in vitro* in the absence of any host cells was unchanged in *fre8*Δ/Δ cells, indicating that there is no primary defect in biofilm formation including adherence. By comparison, *fre8*Δ/Δ biofilms exhibited a consistent increase in killing by neutrophils (Fig 12B). Thus, the *fre8*Δ/Δ defect with *in vivo* biofilms may not reflect a deficit in biofilm formation but rather increased clearance by host immune mechanisms including neutrophils.

We tested whether this increased killing was unique to neutrophils or could be extended to other phagocytes, e.g., macrophages. Our control for macrophage studies was the *sod5*Δ/Δ *C*. *albicans* strain that has been previously shown to be sensitive to macrophage killing due to an inability to degrade host superoxide [11]. We observe that both *sod5*Δ/Δ and *fre8*Δ/Δ mutants show statistically significant increases in killing by bone marrow derived macrophages (BMDM) (Fig 12C). The impact of Fre8 derived ROS appears to extend beyond the hyphal morphology effects seen in fungal-only cultures and interactions with host cells are also important.

## Discussion

NOX enzymes have evolved to intentionally produce ROS, and until recently, were believed to be a property of multicellular differentiation [21, 22]. The discovery of Yno1 in *S*. *cerevisiae* demonstrated that a unicellular fungi can produce ROS through NOX, although the ROS in this case was found to be intracellular [23, 34]. Here we describe *C*. *albicans* Fre8 as the second example of NOX in an organism that can grow as a unicellular yeast and the first for this opportunistic fungal pathogen. Moreover, unlike *S*. *cerevisiae* Yno1, *C*. *albicans* Fre8 is capable of producing extracellular ROS, akin to NOX enzymes in multicellular organisms [1]. In animal cells, NOX enzymes can partner with extracellular SODs that convert the extracellular superoxide free radical to the diffusible H_2_O_2_ molecule [1–5]. Likewise *C*. *albicans* Fre8 appears to partner with extracellular Sod5, providing a rationale for expressing Sod5 only in hyphal cells [7]. These studies also provide a new twist to Sod5 function at the host-pathogen interface. While Sod5 can clearly react with superoxide from macrophage and neutrophil NOX enzymes [11, 12], our studies here with *C*. *albicans* Fre8 indicate that the superoxide for Sod5 is not just coming from the host. It is conceivable that when hyphal cells interact with neutrophils or macrophages, that a “superoxide superstorm” ensues with ROS coming from both the sides of the host-pathogen axis, and with Sod5 operating in the middle.

Why does *C*. *albicans* produce ROS during hyphal morphogenesis? Lessons may be taken from multicellular fungi or fruiting body fungi that use NOX derived ROS for morphogenesis and differentiation [18–20]. For example, ROS from these fungal NOX have been implicated in calcium signaling [58], MAP kinase and Rac1 GTPase signaling [46, 59] and cell re-modeling involving cytoskeleton effects [23, 60]. We show that Fre8 derived H_2_O_2_ can modulate morphogenesis. Work in other systems has shown that NOX derived H_2_O_2_ targets multiple redox sensitive molecules, including protein tyrosine phosphatases, casein kinases with peroxide sensitive degrons; even actin itself can be modulated by oxidation [1, 5, 61, 62]. Since Fre8 ROS is specifically seen at the growing tip of developing hyphae, the H_2_O_2_ produced may act locally on one or more redox sensitive targets that promote polarized growth. The precise mechanism of Fre8 control of hyphal biology is the subject of ongoing investigations.

In addition to the morphology defects of *fre8*Δ/Δ mutants *in vitro* and *in vivo*, we observed that these cells are more sensitive to killing by neutrophils and macrophages *in vitro*. It is possible that the morphological changes in *fre8*Δ/Δ may somehow render these fungal cells more susceptible to attack by phagocytes. As an alternative possibility, the ROS from Fre8 may help condition cells for the oxidative attack by immune cells. It has been proposed that low dose exposures of *C*. *albicans* to H_2_O_2_ or to ROS from macrophages can induce adaptive mechanisms to guard against subsequent oxidative insults [63–66]. *FRE8* ROS may promote such adaptation against neutrophil and macrophage attack. In future studies, it will be important to determine the impact of *fre8*Δ/Δ mutations in immunocompromised settings.

Is Fre8 the only NOX of *C*. *albicans*? This organism has evolved with a very large family of 17 NOX/FRE enzymes, four of which are metalloreductases (including Frp1 characterized here), leaving 12 with unknown functions [35–39]. The extracellular ROS burst studied here is completely eliminated in *fre8*Δ/Δ cells suggesting that Fre8 is the only NOX for extracellular ROS in *C*. *albicans* at least under these *in vitro* conditions. During *C*. *albicans* invasion of the kidney, Fre8 is the most abundantly expressed member of the FRE/NOX family [40]. Even so, it is possible that other members of this family induced during fungal infection and hyphal morphogenesis such as Fre2 (S1 Fig) may similarly function in a NOX capacity, perhaps secondary to Fre8 [40]. *C*. *albicans* may also express NOX enzymes for intracellular ROS analogous to Yno1 of *S*. *cerevisiae*. With such a large family of NOX/FRE enzymes, we speculate that additional NOX enzymes will come to light as mediators of ROS signaling in *C*. *albicans*. Regardless, it will be of interest to integrate Fre8-ROS signaling into known pathways of hyphal regulation.

## Materials and methods

### Yeast strains and growth medium

Cultures of *Candida albicans* cells were typically maintained at 30°C in a yeast extract, peptone based medium (YPD) with 2% (wt/vol) glucose, conditions which support the budding yeast-form of the fungus. The *C*. *albicans fre8*Δ/Δ and *sod5*Δ/Δ strains used in these studies grew identical to WT SC5314 in the yeast-form (S4 Fig). To induce hyphal morphogenesis, yeast-form cells were harvested, starved for 30 min in sterile H_2_O at 30°C, followed by harvesting and induction of hyphal formation by incubating at 37°C (or 34°C, see below) in various media known to stimulate hyphal formation, including Iscove’s Modified Dulbecco’s Medium (IMDM; Gibco), alkaline YPD (50 mM glycine, pH 9.5), spider medium (1% nutrient broth, 1% mannitol, 11.5 mM potassium phosphate, pH 7.2) or YPD with 5–20% fetal bovine serum (heat inactivated, Corning/Cellgro). Hyphal morphogenesis was stimulated in either “low density” (optical density, OD_600_ = 0.1–0.2) or “high density” (OD_600_ = 3.0) conditions. In studies of hyphal morphology, yeast-form cells were cultured to OD_600_ ≈ 8.0, conditions where all cells grew identically (S4 Fig), and were stimulated to form hyphae with YPD-serum. The level of serum used to investigate the *fre8*Δ/Δ defect ranged from 5–15% depending on the lot of serum, with low density cultures typically requiring less serum and temperatures of 34°C to demonstrate a dependence on serum for hyphal morphogenesis. Where indicated, cultures were supplemented with 0.1–10 mUnits glucose oxidase (Type II Sigma#G6125) to bypass the *fre8*Δ/Δ defect in hyphal development. Experiments involving yeast-form cells expressing *FRE8* under the *MET3* promoter used a synthetic complete (SC) based medium containing 0.67% yeast nitrogen base lacking cysteine and either containing or lacking 85.6 mg/L methionine. Cell were seeded at OD_600_ = 0.1 and grown for 1–7 hours.

All *C*. *albicans* strains used in this study were isogenic to SC5314 or its derivative CA-IF100 (*arg4*Δ/*arg4*Δ, *leu2*Δ/*leu2*Δ::*cmLEU2*, *his1*Δ/*his1*Δ::*cdHIS1*, *URA3*/*ura3*Δ). The *sod1Δ*/*Δ*, *sod5*Δ/Δ and *sod4Δ/Δ sod5Δ/Δ sod6Δ/Δ* strains derived from CA-IF100 were kind gifts of Karl Kuchler as previously described [11]. The *cph1Δ/Δ efg1Δ/Δ* strain derived from SC5314 was a gift from Gerald Fink [42]. Mutations in *FRE8* and *SOD5* were introduced in SC5314 using the *SAT1*-flipper cassette method [67]. Deletion in a single *FRE8* allele was achieved using plasmid pJGFRE8LKO, in which *FRE8* regions -926 to -581 and +2402 to +2802 were inserted into the Kpn1 and XhoI and the NotI and SacI sites respectively of pSFS2 [67]. Following liberation of the cassette by KpnI and SacI digestion and transformation of SC5314 by electroporation, accurate deletion of a single *FRE8* allele was verified by PCR, generating the *fre8Δ/+* mutant strain CA-JG201. The second *FRE8* allele was deleted similarly using a pSFS2 construct, pJGFRE8SKO, containing *FRE8* sequences -587 to -3 and +2075 to +2427, creating the *fre8Δ/fre8Δ* strain CA-JG211. Homozygous *sod5*Δ/Δ mutations were introduced in either SC5314 (generating strain CA-JG201) or CA-JG211 (generating CA-JG221) using a construct containing *SOD5*–492 to +53 and +808 to +1253 inserted into the Kpn1 and XhoI and the Not I and SacI sites respectively of pSFS2 [67]. Deletion of both copies of *sod5*Δ/Δ in strain CA-JG201 and CAJG-221 was verified by PCR. A single copy of *FRE8* was introduced into the *fre8*Δ/Δ strain CA-JG211 as follows: *FRE8* sequences -926–+2802 were inserted into the KpnI and XhoI sites of pJGFRE8LKO. Integration into the *FRE8* locus at position -926 to +2802 was achieved by transformation of the cassette liberated by digestion with KpnI and SacI generating the *fre8*Δ/Δ:*FRE8* re-integrant strain CA-JG231. To create the construct for expressing *FRE8* under control of the *MET3* repressible promoter, *C*. *albicans MET3* sequences -1643 to -1 were inserted into Sph1 and Nhe1 sites engineered at *FRE8* position -1 in the pJGFRE8LKO re-integrant plasmid described above. Following digestion with Kpn1 and Sac1, the *MET3-FRE8* containing cassette was used to transform the *cph1Δ/Δ efg1Δ/Δ* strain, the *fre8*Δ/Δ strain CA-JG211 and SC5314 by electroporation. Accurate integration at the *FRE8* locus -926 to +2802 was verified by PCR.

Expression of recombinant *FRE8* and *FRP1* in *Pichia pastoris* used the PichiaPink Strain 1: *ade2* (Thermo Fisher Scientific). *P*. *pastoris* cells were maintained in YP-Gal medium (1% yeast extract, 2% peptone, 2% galactose). Protein expression experiments used a buffered YP medium (1% yeast extract, 2% peptone, 100 mM potassium phosphate, pH 6.0, 1.34% yeast nitrogen base, 0.00004% biotin) that was supplemented with either 0.5% methanol to induce protein expression under the *AOX2* promoter or with 1% glycerol for non-inducing conditions. The plasmid for expressing *FRE8* or *FRP1* under the *P*. *pastoris AOX2* promoter represented a modified version of pPINK α-HC (Thermo Fisher Scientific) in which a 10X HIS tag was introduced downstream of the α-factor pre-sequence (plasmid pRPp718, kind gift of Ryan Peterson). Following the insertion of a Afe1 site downstream of the HIS tag, *FRP1* sequences +1 to +1665 and *FRE8* +1 to +2220 were inserted into the Afe1 and Fse1 sites of this expression plasmid, creating in-frame fusions to the N-terminus secretion sequence and HIS tag. The CTG codons from both genes were altered to TCG for optimal expression in *P*. *pastoris*; plasmids were linearized by digestion with Spe1 and integrated into the *TRP2* locus by transformation.

### Biochemical assays

For luminol and lucigenin measurements of ROS, either yeast-form cells (grown in YPD to OD_600_ of 1.0–2.0) or cells induced to form hyphae as described above were used. Cells were harvested, washed, and suspended in a final OD_600_ of 0.2 in Hanks buffered saline solution (HBSS) containing 0.2 mM luminol (Cayman chemicals) and 0.5 units/ml horseradish peroxidase. In studies with lucigenin, cells were first washed in 25 mM glycine pH 9.5, 0.5% glucose prior to resuspending in 200 μl of the same alkaline buffer containing 5 μM lucigenin. Samples were analyzed for luminol or lucigenin chemiluminescence in 96 well plates using a BioTek Synergy HT plate reader. Analysis was carried out over 1.5 hours at 37°C with a gain setting at 120–135 and integration time of 1.0 second. Results were plotted according to relative luminescence units (RLU) per 0.04 OD_600_ units of cells. With experiments involving DPI, 5 μl of DMSO containing the indicated amount of DPI (or no DPI as control) was added to the reaction at time zero.

For qRT-PCR analysis of fungal-only cultures, 50 ml cultures of cells were induced to form hyphae for 1 hr by growth in YPD-10% FBS (as described above); these early hyphal cells or the control yeast-form were harvested, washed and RNA prepared by the hot acid phenol method [68]. cDNA was prepared using the Maxima H Minus First Strand cDNA Synthesis Kit (ThermoFisher Scientific) and qRT PCR carried out using iTaq Universal SYBR Green Supermix (Bio-Rad). Values were normalized to *TUB2* and graphed according to the fold change in *FRE8* and *SOD5* expression in early hyphal versus yeast-form cells. Amplicons of ≈150 residues were prepared using primers as described in S1 Table.

For analysis of inflammatory mRNA markers (TNF-α, IL-17a, IL-6), RNA from whole kidneys was extracted as previously described [69]. cDNA prepared from 5.0 μg of RNA was diluted 1:50 prior to PCR analysis as above. Values were normalized to ActB and graphed according to the fold change over uninfected controls. Primers for host mRNA analyses are listed in S1 Table.

For ferric reductase and NOX activity analyses in *P*. *pastori* transformants, cells were grown overnight in 10 ml YP-Gal, washed twice in either glycerol or methanol containing buffered media (described above) and resuspended at an OD_600_ of 0.1 in 15 mls of the same medium. Following growth at 30°C for 6 hrs, cells were harvested and washed in either HBSS for the luminol assay or 50 mM citrate, pH 6.6, 5% glucose for the ferric reductase assay. Cells were subjected to luminol chemilumiscence precisely as described above for *C*. *albicans*. Compared to *C*. *albicans* assays, the luminol substrate appears rapidly depleted in *P*. *pastoris* expressing high levels of *FRE8*. For the ferric reductase assay, cells at a OD_600_ of 0.5 were incubated in 200 μl of a reaction containing 1 mM FeCl_3_ and 1 mM bathophenanthrolinedisulfonic acid (BPS) in 50 mM citrate, pH 6.6, 5% glucose. Absorbance at 515 or 520 nm was read in 96 well plates on a BioTek Synergy HT plate reader over 1.5 hours at 30°C. Where designated, 0.1 U of bovine Cu/Zn SOD1 or 50 nM DPI were added to the luminol or ferric reductase assay at t = 0. Ferric reductase measurements in *C*. *albicans* cells was conducted similarly, using cells induced to form hyphae in IMDM for 1 hr as described above and assayed for ferric reductase using the same conditions described for *P*. *pastoris* except *C*. *albicans* cells were assayed at OD_600_ of 0.1.

Total cellular accumulation of copper and iron was measured by inductively coupled plasma mass spectrometry (ICP-MS) using *C*. *albicans* cells induced to form hyphae for 1 hr in 10% FBS as described above. Cells were washed twice with 10 mM Tris, 1 mM EDTA, pH 8 and twice with MiliQ deionized water. Cell pellets containing 10.0 OD_600_ units of cells were resuspended in 500 μl of 20% nitric acid and digested by incubation at 90°C overnight. Samples were diluted 10-fold in MiliQ deionized water and subjected to elemental analysis on a Agilent 7700x ICP-MS instrument.

### *In vitro* biofilm model and fungal killing by neutrophils and macrophages

*In vitro* biofilms were grown in the wells of 96-well microtiter plates, as previously described [70]. Briefly, *C*. *albicans* resuspended in RPMI-MOPS at 1.5 x 10^6^ cells/ml (200μL/well) was added, and incubated for 24 hours at 37°C with 5% CO_2_. To assess biofilm burden an XTT (2,3-Bis-(2-Methoxy-4-Nitro-5-Sulfophenyl)-2H-Tetrazolium-5-Carboxanilide) assay was performed as an estimate of viable burden, as previously described [70].

For assays involving neutrophils, human neutrophils were collected as follows: Blood was obtained from volunteer donors with written informed consent through a protocol approved by the University of Wisconsin Internal Review Board (IRB). Primary human neutrophils were purified by negative antibody selection using the MACSxpress Neutrophil Isolation and MACSxpress Erythrocyte Depletion kits (Miltenyi Biotec Inc., Auburn, CA), as previously described [71]. Experiments with neutrophils were performed in RPMI 1640 (without phenol red) supplemented with 2% heat-inactivated fetal bovine serum (FBS) and glutamine (0.3 mg/ml). Incubations were at 37°C with 5% CO_2_. An adaptation of the XTT metabolic assay was used to estimate *C*. *albicans* viability following co-culture with neutrophils [71]. Following a 24 h incubation period, biofilms were washed with DPBS and neutrophils were added at 1.5 x 10^6^ cells/ml, which represented an effector:target of 1:2. Following a 4 h incubation, 90 μL of 9:1 XTT working solution (0.75 mg/ml XTT in DPBS with 2% glucose: phenazine methosulfate 0.32 mg/ml in ddH2O) was added to each well. After a 25 minutes incubation, samples were transferred to a Falcon 96 well U bottom plate and centrifuged at 1,200×g for three minutes to pellet cells. Supernatants (110 μl) were then transferred to a 96 well flat bottom plate for absorption reading at 492 nm. A neutrophil only control was used to subtract their contribution to the XTT values. To determine percent killing, values were compared to wells without neutrophils after subtraction of the baseline absorbance.

Macrophage infection assays used bone-marrow derived macrophages (BMDM) isolated from the marrow of hind leg bones of 5- to 8-wk-old C57BL-6 female mice. For differentiation, cells were seeded in 100 mm treated cell culture dishes (Corning, Corning, NY) in Dulbecco’s Modified Eagle medium (DMEM; Corning) with 20% L-929 cell-conditioned medium, 10% FBS (Atlanta Biologicals, Flowery Branch, GA), 2mM Glutamax (Gibco, Gaithersburg MD), 1% nonessential amino acids (Cellgro, Manassas, VA), 1% HEPES buffer, 1% penicillin-streptomycin and 0.1% 2-mercaptoethanol for 6–7 days at 37°C with 9.5% CO_2_. 10^5^ BMDM were seeded on 96 well plates and activated by incubating overnight using 100 U/ml of IFN-γ (Roche, Indianapolis, IN). *C*. *albicans* obtained from overnight cultures in YPD (OD_600_ = 8.0) and starved in water as for hyphal morphogenesis studies (see above) were washed twice with PBS and incubated for 30 min at 37°C with Guinea pig complement (MP biomedicals, LLC, OH) for opsonization. The fungus was then added to macrophages at a multiplicity of infection (MOI) ratio of 1:10 for 4 hours. After incubation, the media was removed and macrophages lysed in water. Fungal viability was assessed by the XTT assay according to Pierce et al [72]. The same XTT assay was used to determined fungal viability following farnesol treatment.

### Rodent infection studies

For the murine model of disseminated candidiasis, ten male BALB/c mice (10 weeks old) per strain were inoculated with 2x10^5^ *C*. *albicans* cells of WT SC5314, the *fre8Δ*/*Δ* strain or the *fre8*Δ/Δ strain complemented by *FRE8* by lateral tail vein injection. Moribund mice were sacrificed by CO_2_ asphyxiation and immediately dissected for harvesting kidneys for histology (see below). Fungal burden and host inflammatory markers were analyzed following 48 hours of infection. The spleen and one kidney was processed for CFUs as previously described [69]. The other kidney was placed in 500 μL Trizol and frozen at -80°C for subsequent RNA analyses (see above). Mouse survival was plotted using a log rank test (Mantel Cox) to query any statistical difference.

A jugular vein rat central venous catheter biofilm infection model was used as previously described [73]. Briefly, 24 h following surgical implantation of a jugular venous catheter, *C*. *albicans* at 10^6^ cells/ml was instilled in the catheter lumen and flushed after 6 h. After 24 h biofilm growth period, catheters were harvested and fixed overnight (4% formaldehyde, 1% glutaraldehyde, in PBS). They were then washed with PBS, treated with 1% osmium tetroxide, and washed again. Samples were dehydrated through series of ethanol washes followed by critical point drying and mounted on aluminum stubs. Following sputter coating with platinum, samples were imaged in a scanning electron microscope (LEO 1530) at 3kV.

### Microscopic visualization of *C*. *albicans in vitro* and in infected kidneys

For NBT staining and microscopic analyses of cell morphology, *C*. *albicans* cells were induced to form hyphae as described above using YPD containing 10% FBS (in the case of NBT staining). Cells were harvested, washed once with HBSS and resuspended in 1 ml HBSS containing 0.05% nitroblue tetrazolium (NBT). Following an incubation for 30 min in the dark at 37°C, cells were washed 1X with HBSS, 1X with 70% Ethanol and resuspended in 200 μl 50% Glycerol/HBSS. Cells were visualized by light microscopy at 100x magnification on a Zeiss Axio ImagerA2 microscope. For analysis of hyphal morphogenesis, dark field microscopy of live *C*. *albicans* cells was accomplished using a Nikon Infinity 1 microscope at 40x magnification. Where indicated, enumeration of cells was carried with culture aliquots first passed through 26 gauge and 31 gauge needles to help break up dense aggregates and enhance visualization of individual cells. Passage through these needles did not affect integrity of the individual cells.

To analyze *C*. *albicans* morphology in infected kidneys, freshly harvested kidneys from infected mice were flash frozen in Tissue Tek O.C.T. compound in a dry ice/ethanol bath and were sectioned to 20 μM thickness by cryotome. Tissue slices were adhered to Superfrost Plus Microscope Slides (Fisherbrand Cat. No. 12-550-15) and subjected to Periodic Acid Schiff (PAS) staining by treatment with 0.5% periodic acid (Sigma) for 5 minutes, rinsing briefly with distilled water, then staining 5 minutes with Schiff’s Reagent (Sigma Aldrich). Following a 5 min rinse with water, the mounted tissue was dehydrated using successive 2 min treatments with 50%, 70%, 80%, and twice 95% and 100% ethanol, followed by three 2 min treatment with xylene isomer mixture (Sigma Aldrich) to remove residual ethanol. Cover slips were then mounted with Permount (Fisher) and slides then imaged on a microscope at 40X magnification.

### Ethics statement

All experiments involving animals were approved by the Johns Hopkins University (protocols # MO16M168 and MO15H134) and University of Wisconsin (protocol # DA0031, MV1947) Institutional Animal Care and Use Committees according to guidelines established by the Animal Welfare Act, The Institute of Laboratory Animal Resources Guide for the Care and Use of Laboratory Animals, and the Public Health Service Policy. Experiments involving neutrophils were approved by IRB (protocol #2013 1758) and involved cells isolated from healthy human adult donors in which written informed consent was obtained at the time of blood draw, following the guidelines and approval of the University of Wisconsin-Madison Center for Health Sciences Human Subjects Committee.

## Supporting information

S1 Table Primers used in these studies. (XLSX)

S1 Fig Members of the *C*. *albicans* FRE family induced during hyphal morphogenesis. Expression of the various members of the *FRE* family listed by ORF designation were examined by qRT-PCR as described in *Materials and Methods*. Shown is the fold change in expression after 1 hour stimulation of hyphal morphogenesis by IMDM compared to yeast-form cells (cultured as in Fig 1). Results represent the averages of triplicate cultures. Genes that were previously characterized as cupric or ferric metalloreductases [37–39] or genes induced during fungal invasion of the kidney [40] are indicated by + marks. The *C*. *albicans* orthologue to *S*. *cerevisiae* Yno1 [18] is indicated.
(TIF)

S2 Fig Farnesol, quorum sensing and *fre8*Δ/Δ mutants. (A,B) WT SC5314 and isogenic *fre8*Δ/Δ cells were induced to form hyphae by culturing cells seeded at 4 x 10^6^ cells/ml at 37°C with 5% serum in the presence of the indicated levels of farnesol or methanol vehicle. Following four hours, cells were either (A) photographed or (B) assayed for viability by XTT as described in *Materials and Methods* where results represent the averages of biological triplicates. The decrease in cell mass/viability with 300 and 400 μM farnesol is statistically significant as determined by ANOVA with Tukey post-test; ****p<0.0001. (C) SC5314 cells seeded at 4 x 10^6^ cells/ml (low density) were cultured for four hours at 37°C in YPD supplemented with or without 5% serum or with conditioned 5% serum media derived from high density WT or *fre8*Δ/Δ cultures (6 x 10^7^ cells/ml). The conditioned medium was obtained by removing cells from the high density cultures through centrifugation. Results show that low density SC5314 forms hyphae at 37°C even in the absence of serum (-serum), but hyphal formation is blocked by conditioned medium from high density WT and *fre8*Δ/Δ cultures, indicative of quorum sensing [48]. Photographs are representative of 5–10 images over 2 experimental trials.
(TIF)

S3 Fig Markers of inflammation and fungal burden during disseminated candidiasis. Mice were infected with either *C*. *albicans* WT SC5314 or the isogenic *fre8*Δ/Δ or the *FRE8* complemented *fre8*Δ/Δ (*fre8*Δ/Δ Res) strain by lateral tail vein injection. Following 48 hours of infection, kidney and spleen were harvested and examined for (A) RNA markers of inflammation in the kidney by qRT-PCR as described in *Materials and Methods*, and (B) CFUs. Results are from 7–8 mice from each group. (A) The mRNA levels of the indicated inflammatory markers is shown as a fold change over uninfected controls. In all three infected strains, the increases in TNF- α, IL-6 and IL-17A are statistically significant compared to uninfected controls (****p<0.0001; ***p<0.0007). There is no statistically significant difference between WT and *fre8*Δ/Δ for any samples as determined by a one-way ANOVA with a Tukey post-test. There was a small (<2 fold) increase in expression of IL-17 and IL6 in the *fre8*Δ/Δ RES compared to WT, but the significance of this small variation is uncertain. (B) CFUs are shown as a function of tissue wet weight. The difference between *fre8*Δ/Δ and the *fre8*Δ/Δ strain complemented with *FRE8* (*fre8*Δ/Δ RES) is significant (*p = 0.039). There is no statistically significant difference in CFUs obtained from spleen.
(TIF)

S4 Fig Growth curves of SC5314, *fre8*Δ/Δ and *sod5*Δ/Δ mutants. The indicated yeast strains were seeded at OD_600_ = 0.001 and grown at 30°C in YPD where growth by OD_600_ was either monitored continuously (TOP) or following a 16 hour period (BOTTOM). Results represent the averages of triplicate cultures (TOP) or of two to five experimental trials of hyphal morphogenesis (BOTTOM).
(TIF)
