# Supplementary figures and images for: Candida albicans FRE8 encodes a member of the NADPH oxidase family that produces a burst of ROS during fungal morphogenesis
Source: PLoS Pathog. 2017 Dec 1;13(12):e1006763. doi: 10.1371/journal.ppat.1006763 (PMC5728582; doi:10.1371/journal.ppat.1006763)

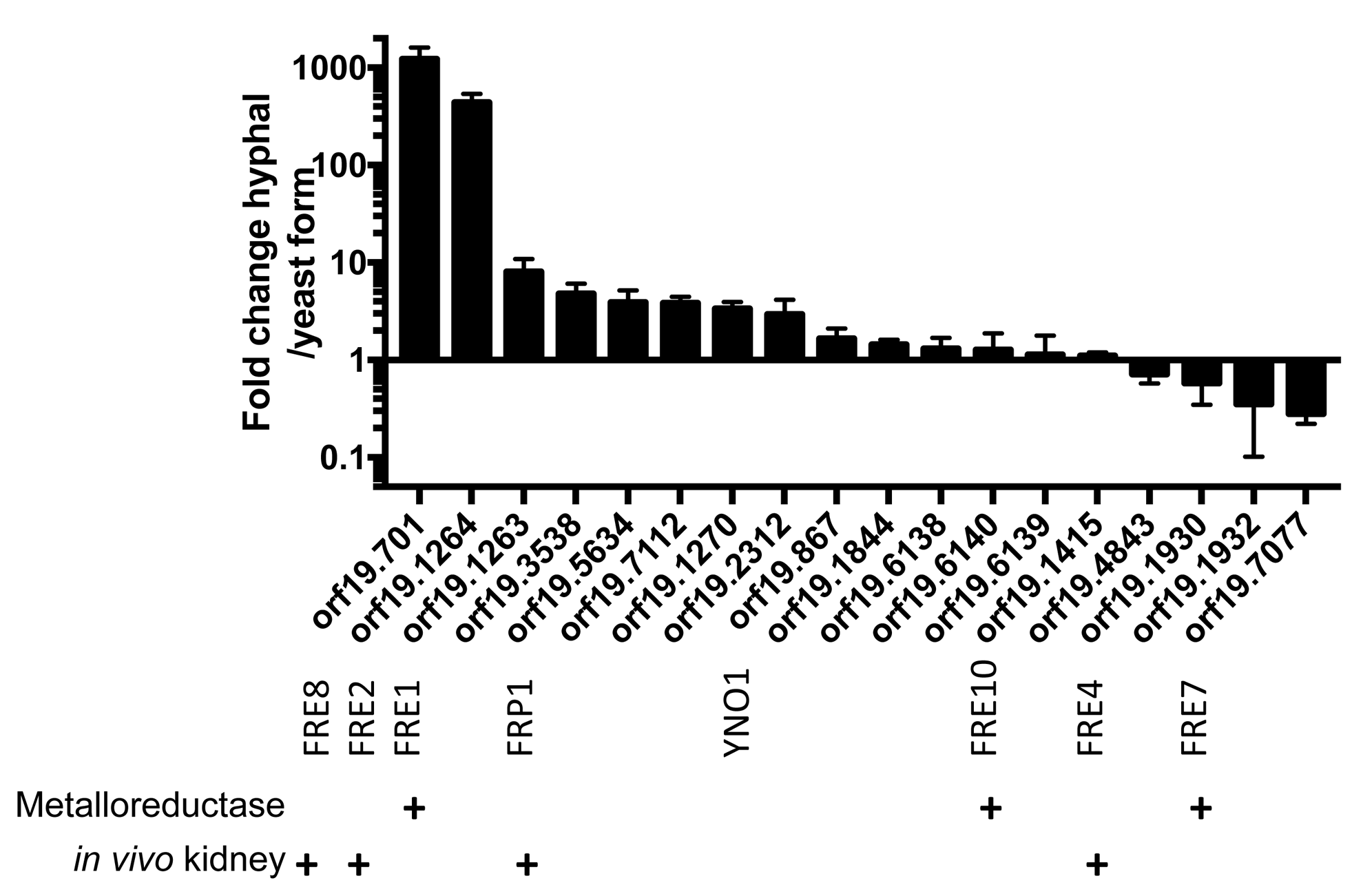

Supplement: S1 Fig — Expression of the various members of the FRE family listed by ORF designation were examined by qRT-PCR as described in Materials and Methods. Shown is the fold change in expression after 1 hour stimulation of hyphal morphogenesis by IMDM compared to yeast-form cells (cultured as in Fig 1). Results represent the averages of triplicate cultures. Genes that were previously characterized as cupric or ferric metalloreductases [37–39] or genes induced during fungal invasion of the kidney [40] are indicated by + marks. The C. albicans orthologue to S. cerevisiae Yno1 [18] is indicated. (TIF) [file ppat.1006763.s002.tif]

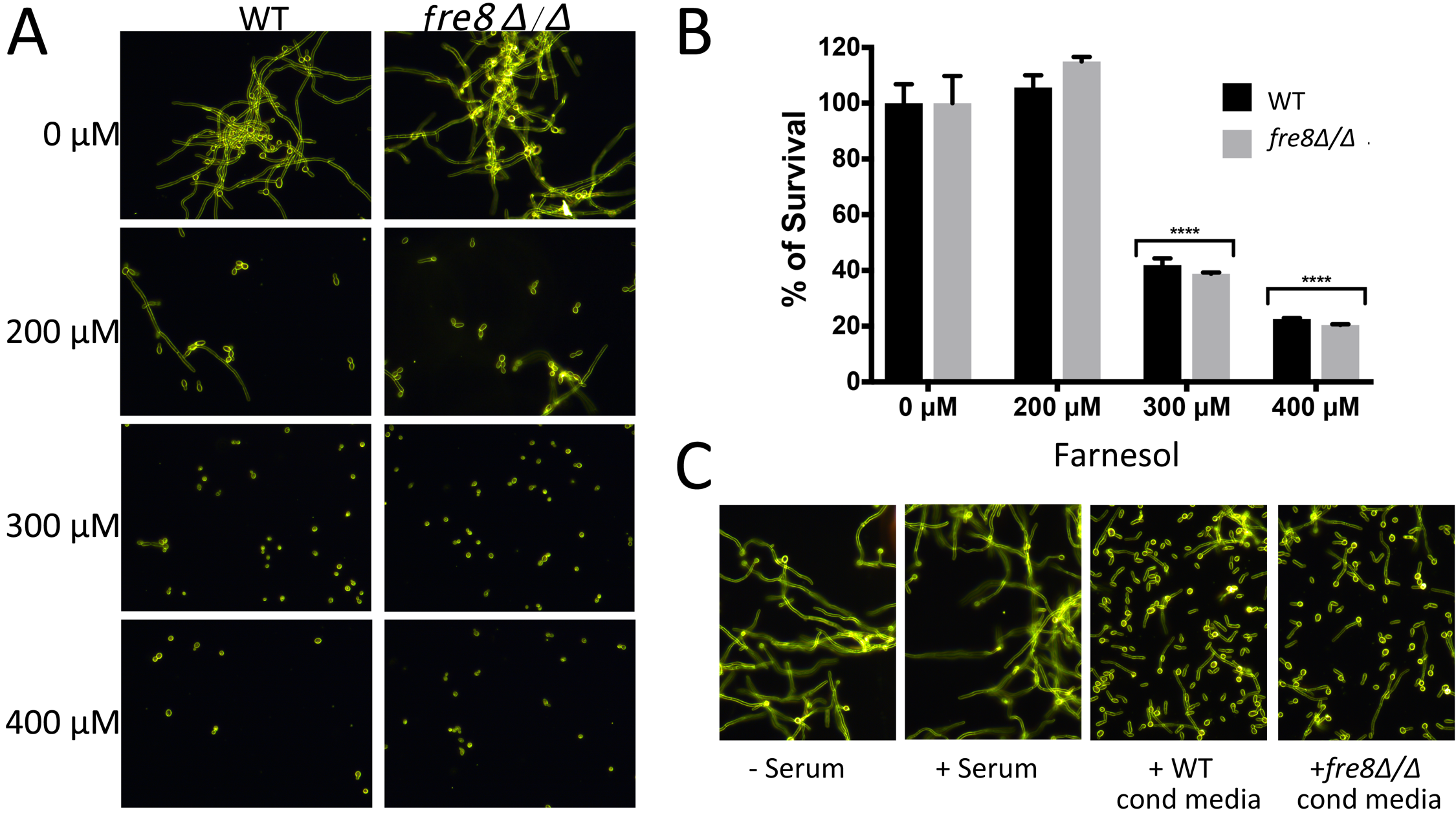

Supplement: S2 Fig — (A,B) WT SC5314 and isogenic fre8Δ/Δ cells were induced to form hyphae by culturing cells seeded at 4 x 10^6^ cells/ml at 37°C with 5% serum in the presence of the indicated levels of farnesol or methanol vehicle. Following four hours, cells were either (A) photographed or (B) assayed for viability by XTT as described in Materials and Methods where results represent the averages of biological triplicates. The decrease in cell mass/viability with 300 and 400 μM farnesol is statistically significant as determined by ANOVA with Tukey post-test; ****p<0.0001. (C) SC5314 cells seeded at 4 x 10^6^ cells/ml (low density) were cultured for four hours at 37°C in YPD supplemented with or without 5% serum or with conditioned 5% serum media derived from high density WT or fre8Δ/Δ cultures (6 x 10^7^ cells/ml). The conditioned medium was obtained by removing cells from the high density cultures through centrifugation. Results show that low density SC5314 forms hyphae at 37°C even in the absence of serum (-serum), but hyphal formation is blocked by conditioned medium from high density WT and fre8Δ/Δ cultures, indicative of quorum sensing [48]. Photographs are representative of 5–10 images over 2 experimental trials. (TIF) [file ppat.1006763.s003.tif]

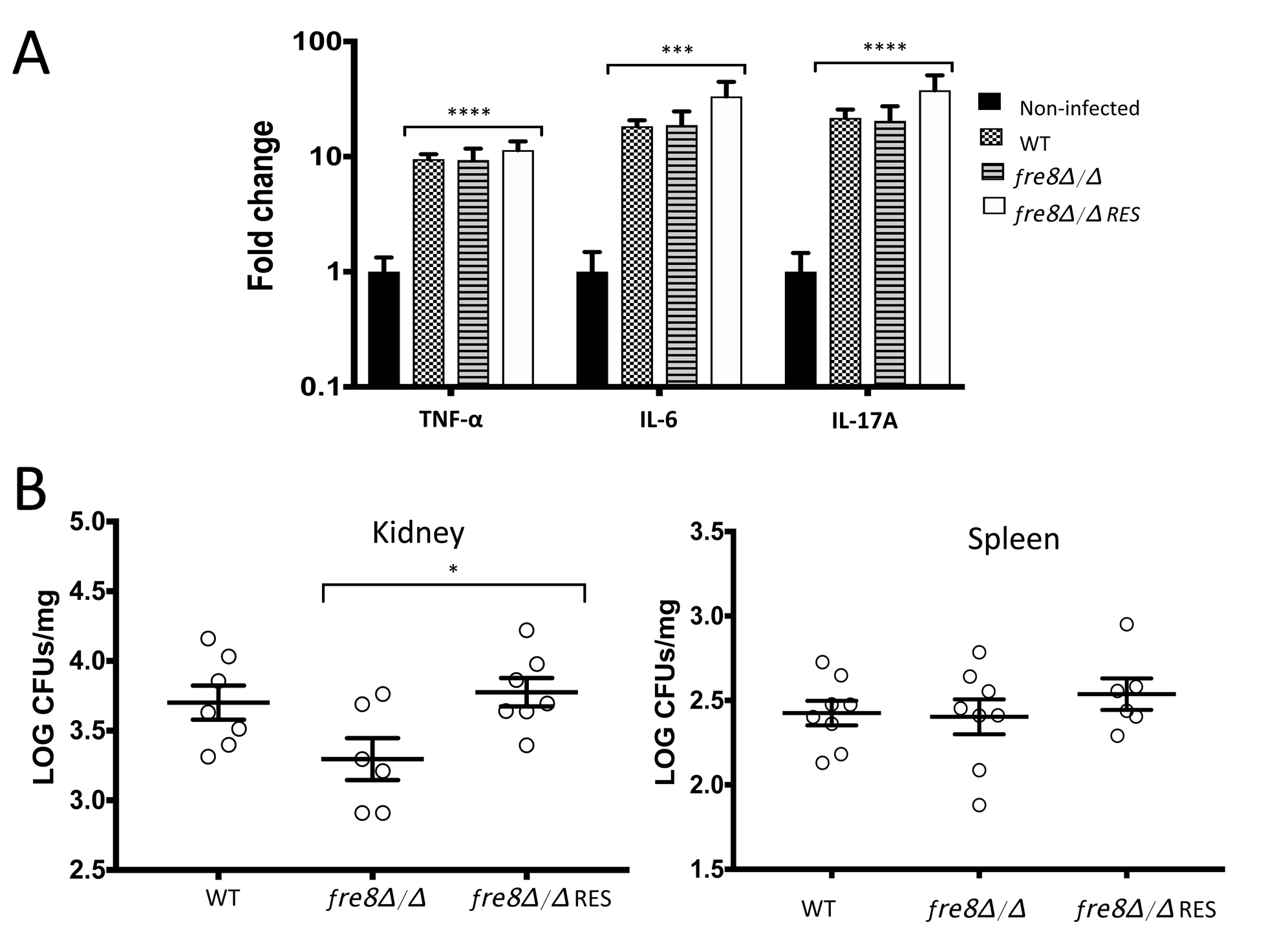

Supplement: S3 Fig — Mice were infected with either C. albicans WT SC5314 or the isogenic fre8Δ/Δ or the FRE8 complemented fre8Δ/Δ (fre8Δ/Δ Res) strain by lateral tail vein injection. Following 48 hours of infection, kidney and spleen were harvested and examined for (A) RNA markers of inflammation in the kidney by qRT-PCR as described in Materials and Methods, and (B) CFUs. Results are from 7–8 mice from each group. (A) The mRNA levels of the indicated inflammatory markers is shown as a fold change over uninfected controls. In all three infected strains, the increases in TNF- α, IL-6 and IL-17A are statistically significant compared to uninfected controls (****p<0.0001; ***p<0.0007). There is no statistically significant difference between WT and fre8Δ/Δ for any samples as determined by a one-way ANOVA with a Tukey post-test. There was a small (<2 fold) increase in expression of IL-17 and IL6 in the fre8Δ/Δ RES compared to WT, but the significance of this small variation is uncertain. (B) CFUs are shown as a function of tissue wet weight. The difference between fre8Δ/Δ and the fre8Δ/Δ strain complemented with FRE8 (fre8Δ/Δ RES) is significant (*p = 0.039). There is no statistically significant difference in CFUs obtained from spleen. (TIF) [file ppat.1006763.s004.tif]

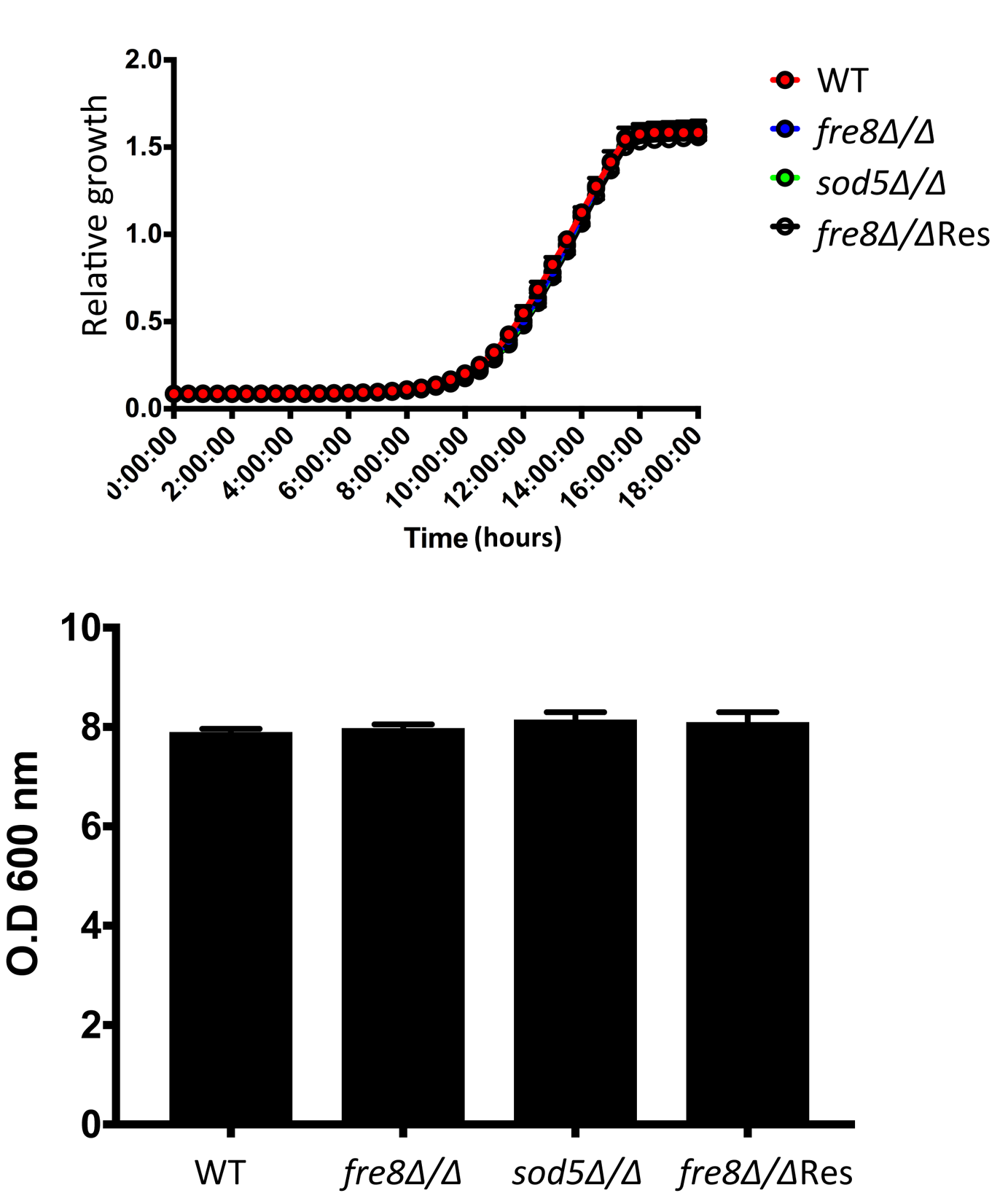

Supplement: S4 Fig — The indicated yeast strains were seeded at OD600 = 0.001 and grown at 30°C in YPD where growth by OD600 was either monitored continuously (TOP) or following a 16 hour period (BOTTOM). Results represent the averages of triplicate cultures (TOP) or of two to five experimental trials of hyphal morphogenesis (BOTTOM). (TIF) [file ppat.1006763.s005.tif]
